# Metabolomics-based comparison of metabolite profiles in *Capparis spinosa* L. from different habitats and associated soil factors

**DOI:** 10.3389/fpls.2026.1914134

**Published:** 2026-10-14

**Authors:** Xiakela Xiayifu, Ruli Zhao, Ziyi Bai, Xiaoying Liu, Xinhui Wang

**Affiliations:** 1 College of Ecology and Environment, Xinjiang University, Urumqi, Xinjiang, China; 2 College of Life Science and Technology, Xinjiang University, Urumqi, Xinjiang, China

**Keywords:** *Capparis spinosa* L., different habitats, metabolomics, quality differences, rhizosphere soil

## Abstract

**Introduction:**

Capparis spinosa L. is a distinctive plant with edible and medicinal value, yet the factors influencing the accumulation of its effective components across diverse habitats remain unclear. This study aims to profile fruit metabolites and explore the association with rhizosphere soil environments.

**Methods:**

Using LC-MS/MS-based untargeted metabolomics (six biological replicates per habitat), fruit metabolites from three habitats in northern Xinjiang (Urumqi, Karamay, and Ili) were profiled. Partial least squares discriminant analysis (PLS-DA) identified differential metabolites. 16S rRNA sequencing analyzed rhizosphere soil microbial communities, and correlation analyses linked fruit metabolites with soil metabolites and microbiota.

**Results:**

A total of 1,739 metabolites were identified. PLS-DA revealed clear habitat separation, with 454, 340, and 411 differential metabolites in pairwise comparisons. Sixteen anti-inflammatory compounds (flavonoids, terpenoids, etc.) were candidate quality-associated metabolites, predominantly highest in Urumqi. Soil bacterial/fungal composition differed significantly among habitats, with Rubrobacterand Gaiella enriched in Urumqi. Correlation analysis linked 16 fruit metabolites (e.g., resveratrol, kaempferol) to 10 soil metabolites and specific soil genera.

**Discussion:**

Habitat-driven differences in soil microbial communities and metabolite levels are associated with bioactive component abundance in C. spinosa fruit, offering targets for habitat selection and microbiome-informed cultivation. Note that LC-MS/MS signal intensities are semi-quantitative; reported differences reflect relative abundance rather than absolute content.

## Highlights

- Habitat is associated with differences in the metabolite profile of the medicinal plant *Capparis spinosa* L.
- Geographical distance leads to the differentiation of the soil microbial communities of the medicinal plant *C. spinosa*.
- The soil microenvironment is associated with soil nutrient levels, which may be linked to relative differences in the quality-related metabolites of *C. spinosa*.

## 1 Introduction

Medicinal plants are a major natural source of drugs, and their active components are central to quality evaluation (Venkatasai et al., 2025). *Capparis spinosa* L. is a desert-adapted, edible medicinal plant distributed across Western Asia, Europe, North America, and Oceania, and mainly in Xinjiang, Tibet, and Gansu within China (Hu et al., 2017; Hassan et al., 2022). Its fruit and stem contain flavonoids, alkaloids, glycosylated metabolites, and other bioactive compounds with anti-inflammatory, anti-hypertensive, and hypoglycemic effects (Aichi-Yousfi et al., 2016; Hu et al., 2017; Amal et al., 2019; He et al., 2023; Huseini et al., 2013; Gianguzzi et al., 2019).

Habitat conditions shape these active components: comparisons across Sardinia, Italy, Morocco, and Turkey have shown that the glucosinolates, phenolic compounds, and flavonoids in *C. spinosa* vary markedly by region (Nowruzian and Aalami, 2025; Azzurra et al., 2018), indicating that geographic and environmental variations are associated with differences in the fruit metabolite profiles.

Underlying such variations, non-biological habitat factors—water availability, temperature, and soil properties—alter plant secondary metabolism (Li et al., 2020; Ma et al., 2016; Tripathi et al., 2025; Naghiloo et al., 2012; Abreu and Mazzafera, 2005), while the soil itself supplies the nutrients and hosts the microbial communities that shape medicinal plant quality: soil organic matter and nutrient status are correlated with the active component levels in other Chinese medicinal plants (Yang et al., 2020; Liu et al., 2023), and soil microorganisms, which differ across regions (Pérez et al., 2026; You et al., 2018; Xie et al., 2025), drive nutrient cycling and influence medicinal plant metabolite formation (Gao et al., 2026). This suggests a chain running from habitat to the soil metabolome and microbiome to the fruit metabolome. However, this chain has not been directly examined in *C. spinosa*.

Research linking *C. spinosa* fruit quality to soil metabolites and microorganisms remains scarce. In this study, LC-MS/MS-based metabolomics and 16S rRNA/internal transcribed spacer (ITS) sequencing were used to profile the fruit and rhizosphere soil metabolites and the microbial communities across three major producing areas in northern Xinjiang, tracing how habitat differences propagate through the soil metabolome and microbiome to shape fruit quality-related metabolites. Specifically, we tested the following hypotheses: 1) habitat significantly alters the relative abundance of the quality-associated metabolites in *C. spinosa* fruit; 2) these habitat-associated differences in fruit metabolites are linked to habitat-specific soil metabolite profiles and the rhizosphere microbial community composition; and 3) specific soil microbial taxa are significantly correlated with the relative levels of key fruit bioactive components, pointing to candidate microbial drivers of fruit quality. Testing these hypotheses is intended to provide a scientific basis for the selection of high-quality production areas, the optimization of soil nutrients, and the development of microbiome-based strategies for the sustainable cultivation of *C. spinosa*, a unique fruit and vegetable resource.

Morphological illustrations of *C. spinosa* L. from the three regions, along with selected field visit photographs, are shown in **Supplementary Figure S1**.

## 2 Results

### 2.1 Analysis of the differential metabolites in *C. spinosa* fruits from different habitats

Based on the results of the metabolomic analysis, and taking into account the metabolites identified from both positive and negative ions modes, a total of 1,739 metabolites were identified in the fruits from the three different habitats. Principal component analysis (PCA) of the Chinese prickly ash fruit samples and the quality control (QC) samples was conducted to examine the distribution of fruit metabolites from Urumqi, Karamay, and Ili. Ion flowcharts of the major peaks were generated for each of the six biological replicates in both positive and negative ion modes (**Supplementary Figures S2A, B**). To reveal differences in the metabolites of *C. spinosa* fruits across different habitats, partial least squares discriminant analysis (PLS-DA) was performed to establish the relationship between the fruit samples and metabolite expression levels in various habitats. The results demonstrated significant separation among sample pairs Urumqi fruits (UF) *vs*. Karamay fruits (KF), UF *vs*. Ili fruits (IF), and KF *vs*. IF in both positive and negative ion modes (**Figure 1A**; **Supplementary Figure S3A**). In both ion modes, the condition *R*^2^*Y* > *Q*^2^*Y* was satisfied, confirming that the model was well established and could effectively characterize the metabolic differences among different habitats. In the comparison of UF *vs*. KF, a total of 454 differential metabolites were identified, with 237 detected in the positive ion mode: 145 were upregulated and 92 were downregulated. For UF *vs*. IF, 340 differential metabolites were identified, of which 213 were detected in the positive ion mode: 183 were upregulated and 30 were downregulated (**Figure 1B**). In the comparison of KF *vs*. IF, 411 differential metabolites were identified, with 196 detected in the positive ion mode: 145 were upregulated and 51 were downregulated. In the comparison between UF and KF, 454 differentially expressed metabolites were identified, of which 217 were detected in negative ion mode: 162 were upregulated and 55 were downregulated. In the comparison between UF and IF, 340 differentially expressed metabolites were identified, of which 127 were detected in negative ion mode: 81 were upregulated and 46 were downregulated. In the comparison between KF and IF, a total of 411 differentially expressed metabolites were identified, of which 215 were detected in negative ion mode: 68 were upregulated and 147 were downregulated (**Supplementary Figure S3B**).

**Figure 1:**
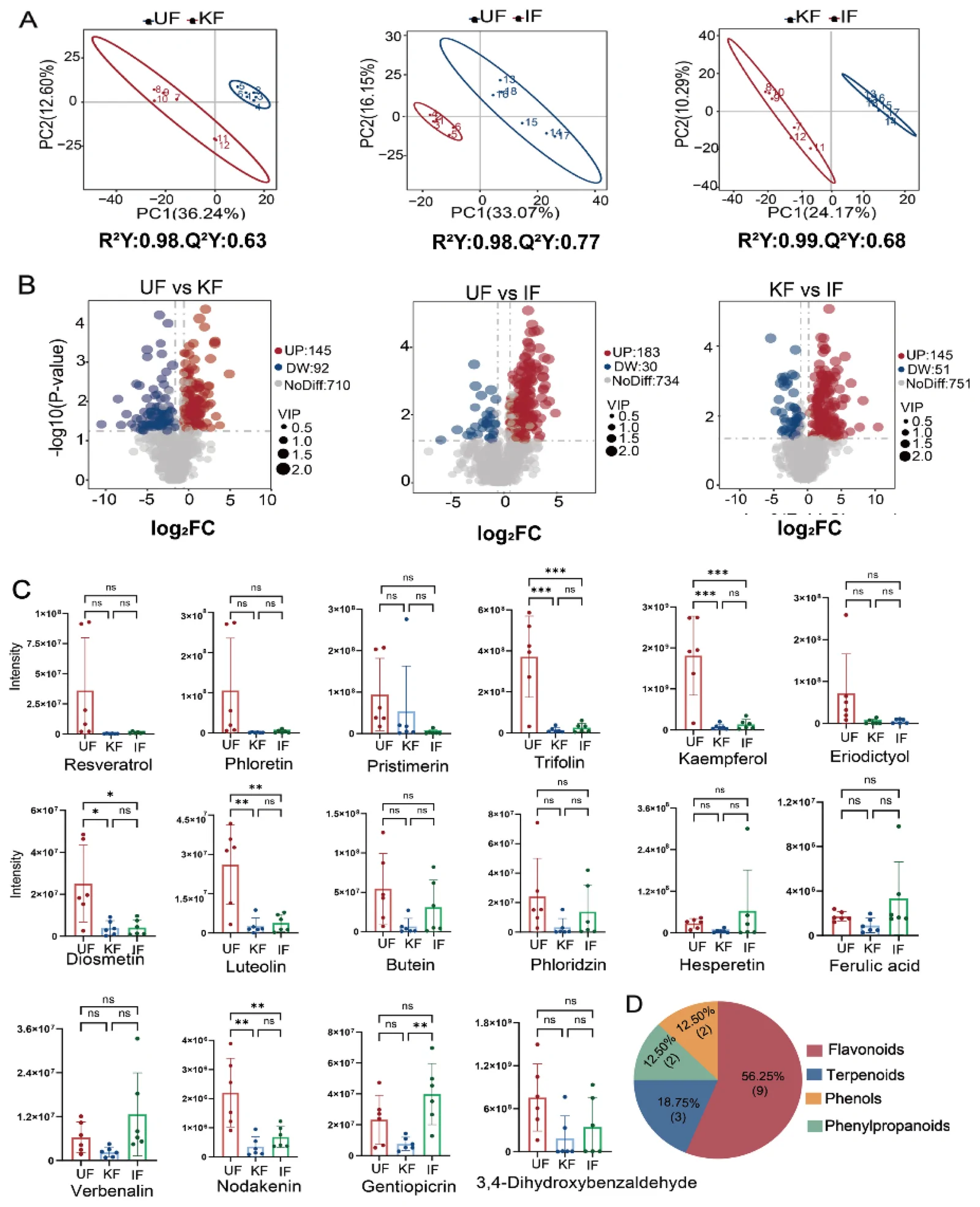
Analysis of the differential metabolites in the fruits of *Capparis spinosa* from different habitats (positive ion mode). *UF*, Urumqi fruit; *KF*, Karamay fruit; *IF*, Ili fruit. *p < 0.05; **p < 0.01; ***p < 0.001. **(A)** PCA score plots showing the separation among UF, KF, and IF groups. **(B)** OPLS-DA volcano plots displaying differential metabolites (red: upregulated; blue: downregulated; gray: non-significant). **(C)** Relative intensity of selected differential metabolites across UF, KF, and IF. **(D)** Pie chart illustrating the classification of differential metabolites (Flavonoids, Terpenoids, Phenols, Phenylpropanoids).

Based on the log_2_FC values of the differential metabolites and searches in the medical library, 16 differential metabolites with anti-inflammatory potential in the fruits were selected as the main effective components of *C. spinosa*, and their distribution across different habitats was investigated (**Figure 1C**). A total of 12 significantly differential metabolites were detected across groups, with six of them (trifolin, kaempferol, diosmetin, luteolin, nodakenin, and gentiopicrin) exhibiting the highest accumulation in UF, all reaching statistical significance (*p* < 0.05, with kaempferol showing *p* < 0.001). The relative levels of gentiopicrin, verbenalin, hesperetin, and ferulic acid were highest in IF. Notably, only the gentiopicrin relative level in IF was higher than that in KF (*p* < 0.01). According to database annotations, the 16 anti-inflammatory-focused differential metabolites primarily consisted of flavonoids, terpenoids, phenols, and phenylpropanoids (**Figure 1D**). The corresponding figures showing the substitution test results and model validation parameters in the positive and negative ion modes are provided in **Supplementary Figures S4A, B**.

### 2.2 Analysis of the differential metabolites in the soil of the different habitats of *C. spinosa*

For six biological replicates of soil samples from different habitats of *C. spinosa*, base peak ion current diagrams of the six major peaks were plotted in both positive and negative ion modes (**Supplementary Figures S5A, B**). A total of 1,600 compounds were identified in the total metabolites of the soils from the three habitats. Analysis of the differences in the soil metabolites around the roots of *C. spinosa* across different habitats revealed significant differences between the Urumqi soil (US), Karamay soil (KS), and Ili soil (IS) groups in both positive and negative modes (**Figure 2A** **Supplementary Figure S6A**). Furthermore, as shown in **Figure 2B**, a total of 714 differential metabolites were identified in the comparison of US *vs*. KS. Among them, 419 differential metabolites were found in the positive ion mode, with 183 upregulated and 236 downregulated. A total of 300 differential metabolites were identified in the comparison of US *vs*. IS. Among them, 194 differential metabolites were found in the positive ion mode, with 114 upregulated and 80 downregulated. A total of 411 differential metabolites were identified in the comparison of KS *vs*. IS. Among them, 196 differential metabolites were found in the positive ion mode, with 145 upregulated and 51 downregulated. A total of 217 differentially expressed metabolites were detected in negative ion mode, comprising 162 upregulated and 55 downregulated metabolites. In the comparison between US and IS, a total of 300 differentially expressed metabolites were identified. Of these, 106 differentially expressed metabolites were detected in negative ion mode, comprising 86 upregulated and 20 downregulated metabolites. In the comparison between KS and IS, a total of 411 differentially expressed metabolites were identified. Of these, 215 differentially expressed metabolites were detected in negative ion mode, comprising 68 upregulated and 147 downregulated metabolites (**Supplementary Figure S6B**). The corresponding figures showing the substitution test results and model validation parameters in the positive and negative ion modes are provided in **Supplementary Figures S7A, B**.

**Figure 2:**
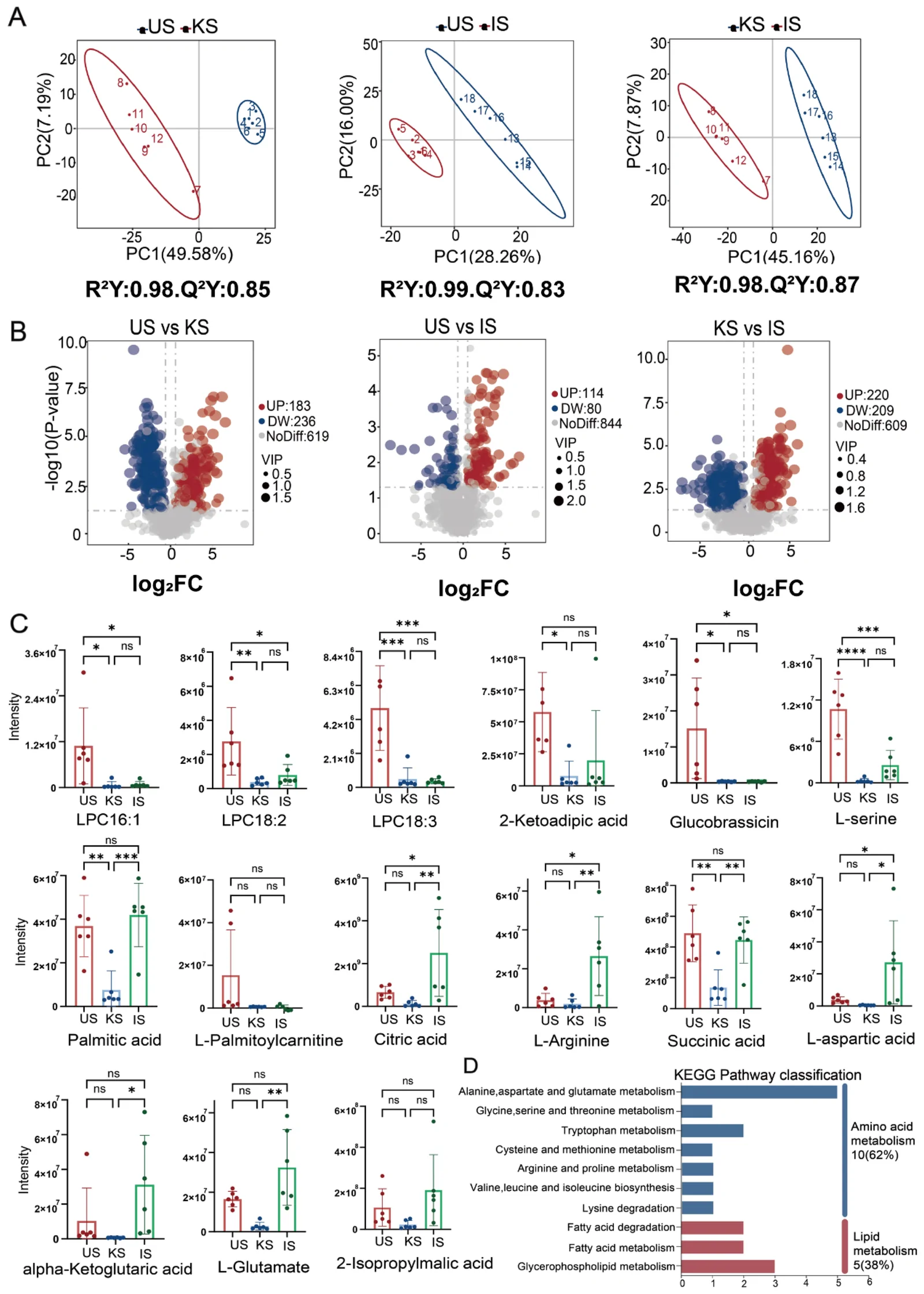
Analysis of the differential metabolites in the soil of the different habitats of *Capparis spinosa* (positive ion mode). *US*, Urumqi soil; *KS*, Karamay soil; *IS*, Ili soil. *p < 0.05; **p < 0.01; ***p < 0.001. **(A)** PCA score plots showing the separation among US, KS, and IS groups. **(B)** OPLS-DA volcano plots displaying differential metabolites (red: upregulated; blue: downregulated; gray: non-significant). **(C)** Relative intensity of selected differential metabolites across US, KS, and IS. **(D)** KEGG pathway classification bar chart of the differential metabolites.

According to the log_2_FC value and the Kyoto Encyclopedia of Genes and Genomes (KEGG) pathway enrichment results, the top 15 differential metabolites enriched in the amino acid metabolism and fatty acid metabolism pathways in soil metabolites were selected (**Figure 2D**), and their distribution in the different habitats was discussed. Among them, eight soil metabolites (glucobrassicin, l-serine, 2-ketoadipic acid, succinic acid, l-palmitoylcarnitine, LPC16:1, LPC18:3, and LPC18:2) had the highest relative levels in the soil of Urumqi (*p* < 0.05), except for l-palmitoylcarnitine. The remaining seven differential soil metabolites (l-aspartic acid, alpha-ketoglutaric acid, citric acid, l-arginine, l-glutamate, 2-isopropylmalic acid, and palmitic acid) had higher levels in IS (*p* < 0.05), except for 2-isopropylmalic acid (**Figure 2C**).

The significant enrichment of the differential metabolites in the amino acid and fatty acid metabolism pathways reflects the adaptive characteristics of the soil microbial communities to distinct environments in Urumqi, Ili, and Xinjiang and holds important biological significance for soil nutrient cycling and ecological function (Mauceri et al., 2024). For amino acid metabolism, the higher levels of l-aspartic acid, l-arginine, and l-glutamic acid in IS indicate a more active nitrogen cycle. At the same time, these amino acids assist microorganisms in coping with abiotic stresses such as salinity and drought, regulating cellular osmotic pressure, and maintaining community communication (Asghari et al., 2020). In terms of fatty acid metabolism, the higher palmitic acid content in IS helps microorganisms enhance the cell membrane stability to adapt to environmental stresses such as temperature fluctuations, while the higher contents of carnitine and lysophosphatidylcholine in US reflect stronger fatty acid transport and β-oxidation processes, which are associated with differences in the organic substrates and microbial community composition between the two regions (Ming et al., 2013; Zhao et al., 2010). Overall, the differences in these two metabolic pathways reflect the adaptation of the microorganisms to different habitats and may be linked to soil fertility maintenance and ecosystem function.

### 2.3 The impact of different habitats on the diversity and species differences of soil bacteria

To elucidate the effects of the different habitats on the soil bacterial communities, the diversity and community variations of the soil bacteria across various habitats were analyzed. According to the alpha diversity index analysis (**Figure 3A**), the soil bacterial diversity and species richness followed the order Urumqi soil bacteria (USB) > Ili soil bacteria (ISB) > Karamay soil bacteria (KSB), with USB exhibiting the highest levels. Notably, the bacterial diversity and richness of USB were higher than that of KSB (*p* < 0.05). The Venn diagram of the operational taxonomic units (OTUs) revealed that the common OTUs among the three habitats accounted for 1.6% of the total OTUs. In addition, the principal coordinates analysis (PCoA) results demonstrated significant separation of the soil samples from the different habitats (**Figures 3B, C**).

**Figure 3:**
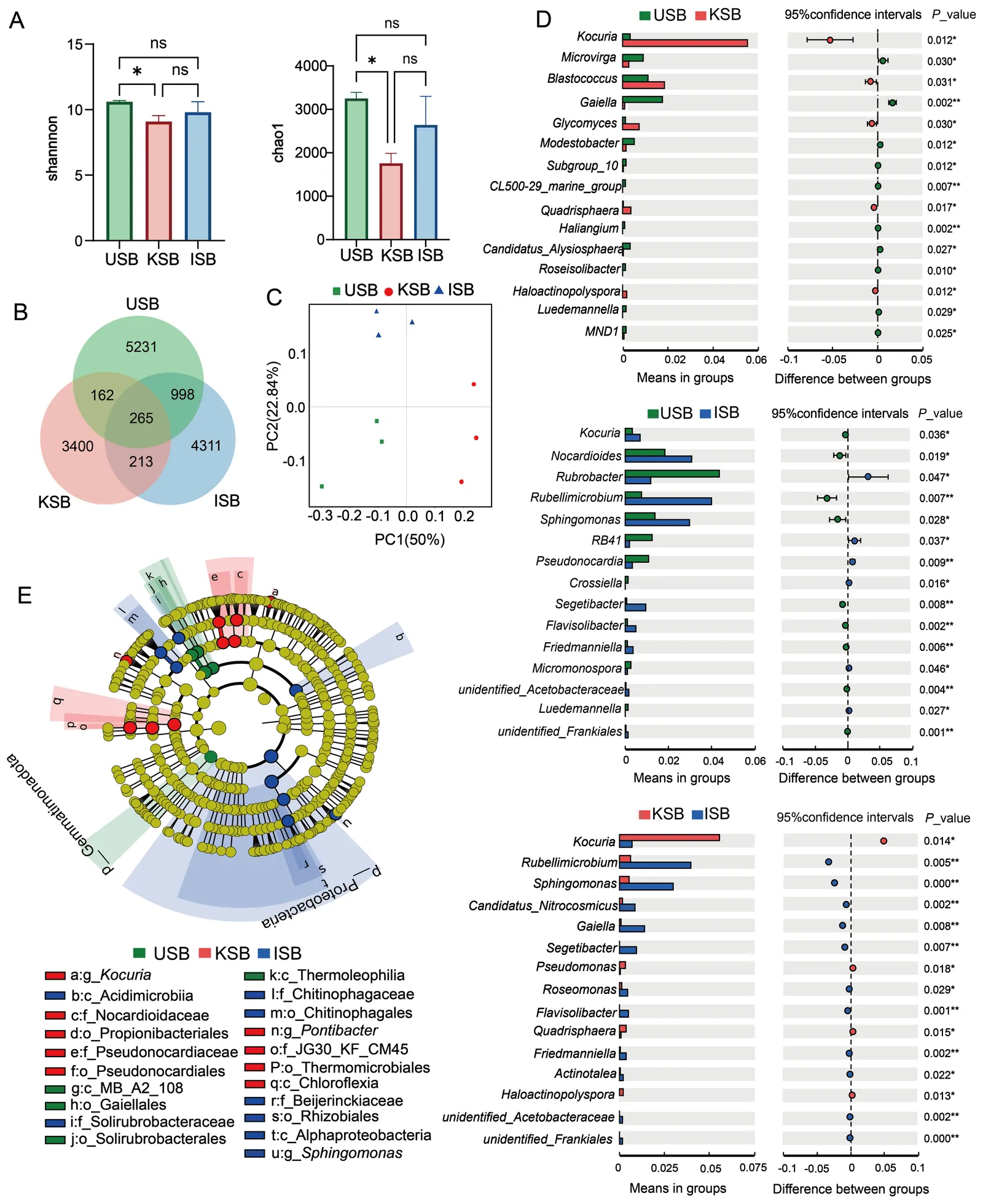
Bacterial diversity and species differences in the different habitats. *USB*, Urumqi soil bacteria; *KSB*, Karamay soil bacteria; *ISB*, Ili soil bacteria. *p < 0.05; **p < 0.01; ***p < 0.001. **(A)** Alpha diversity among USB, KSB, and ISB groups (ns: not significant). **(B)** Venn diagram showing the shared and unique OTUs/ASVs among the three groups. **(C)** PCoA plot based on Bray-Curtis distance displaying the separation of the groups. **(D)** Differential abundance analysis of bacterial genera between groups with 95% confidence intervals and P-values. **(E)** Circos plot illustrating the community composition and interactions across different taxonomic levels and habitats.

Regarding community composition, the soil bacterial communities in the different habitats were predominantly composed of Actinobacterota, Proteobacteria, Chloroflexi, and Bacteroidota (**Supplementary Figure S8A**). The genus-level *t*-test analyses (**Figure 3D**) showed that, when comparing USB and KSB, 10 bacterial genera (*Microvirga*, *Gaiella*, *Modestobacter*, *Subgroup_10*, *Haliangium*, *Roseisolibacter*, *CL500-29_marine_group*, *Candidatus Alysiosphaera*, *Luedemannella*, and *MND1*) had higher abundance rates in USB (*p* < 0.05), whereas the remaining differential genera were more abundant in KSB (*p* < 0.05). In the comparison between USB and ISB, six genera (*Rubrobacter*, *Pseudococcus*, *Micromonospora*, *Crossiella*, *RB41*, and *Luedemannella*) were more abundant in USB (*p* < 0.05), while nine genera showed significantly higher abundance rates in ISB (*p* < 0.05). For the KSB *vs*. ISB comparison, four genera (*Kocuria*, *Pseudomonas*, *Quadrisphaera*, and *Haloactinopolyspora*) were more abundant in KSB (*p* < 0.05), while 11 genera had higher abundance in ISB (*p* < 0.05). Based on the linear discriminant analysis effect size (LEfSe) across the phylum, class, order, and genus levels (**Figure 3E**), Gemmatimonadota, MBA_A2_108, Thermolophilia, Gaiellales, and Solirubrobacterales were identified as the biomarkers for USB. Chloroflexia, Propionibacterium, Pseudochondriales, Thermomicrobiales, *Kocuria*, and *Pontibacter* were enriched in KSB, while Proteobacteria, Acidemebiia, Alphaproteobacteria, Chitinophagales, Rhizobiales, and *Sphingomonas* were specifically enriched in ISB.

### 2.4 The impact of different habitats on the diversity and species differences of soil fungi

Analysis of the fungal alpha diversity across the different habitats revealed that the Shannon and Chao1 index values followed the order Ili soil fungi (ISF) > Karamay soil fungi (KSF) > Urumqi soil fungi (USF), indicating higher fungal diversity in ISF. For species richness, the ranking was ISF > USF > KSF. Notably, the fungal diversity of ISF was significantly higher than that of USF (*p* < 0.05) (**Figure 3A**). A Venn diagram of the fungal communities (**Figure 3B**) showed that only 1.6% of the fungal OTUs were common among the three habitats. The PCoA results further demonstrated significant separation of the soil samples from the different habitats, highlighting distinct fungal community profiles (**Figure 4C**). In terms of community composition, the soil fungal communities across habitats were predominantly composed of the Ascomycota and Basidiomycota phyla (**Supplementary Figure S8B**). The genus-level *t*-test analyses (**Figure 4D**) showed that, when comparing USF and KSF, *Alternaria* and *Filobasidium* had significantly higher abundance rates in KSF, while the remaining fungal genera were more abundant in USF (*p* < 0.05). In the USF *vs*. ISF comparison, *Filobasidium* was more abundant in ISF, whereas *Spizellomycetaceae_gen_incertae_sedis* and *Chaetomium* were more abundant in USF (*p* < 0.05). When comparing KSF and ISF, *Alternaria* and *Filobasidium* were more abundant in KSF, while *Oedocephalum* showed higher abundance in ISF (*p* < 0.05). The LEfSe results at the phylum, class, and order levels (**Figure 4E**) identified Sordariomycetes and *Clitopilus* as specific biomarkers for USF. Dothideomycetes, Pleosporales, *Alternaria*, *Heydenia*, and *Filobasidium* were specifically enriched in KSF, whereas Tremellomycetes and Phialophora were enriched in ISF.

**Figure 4:**
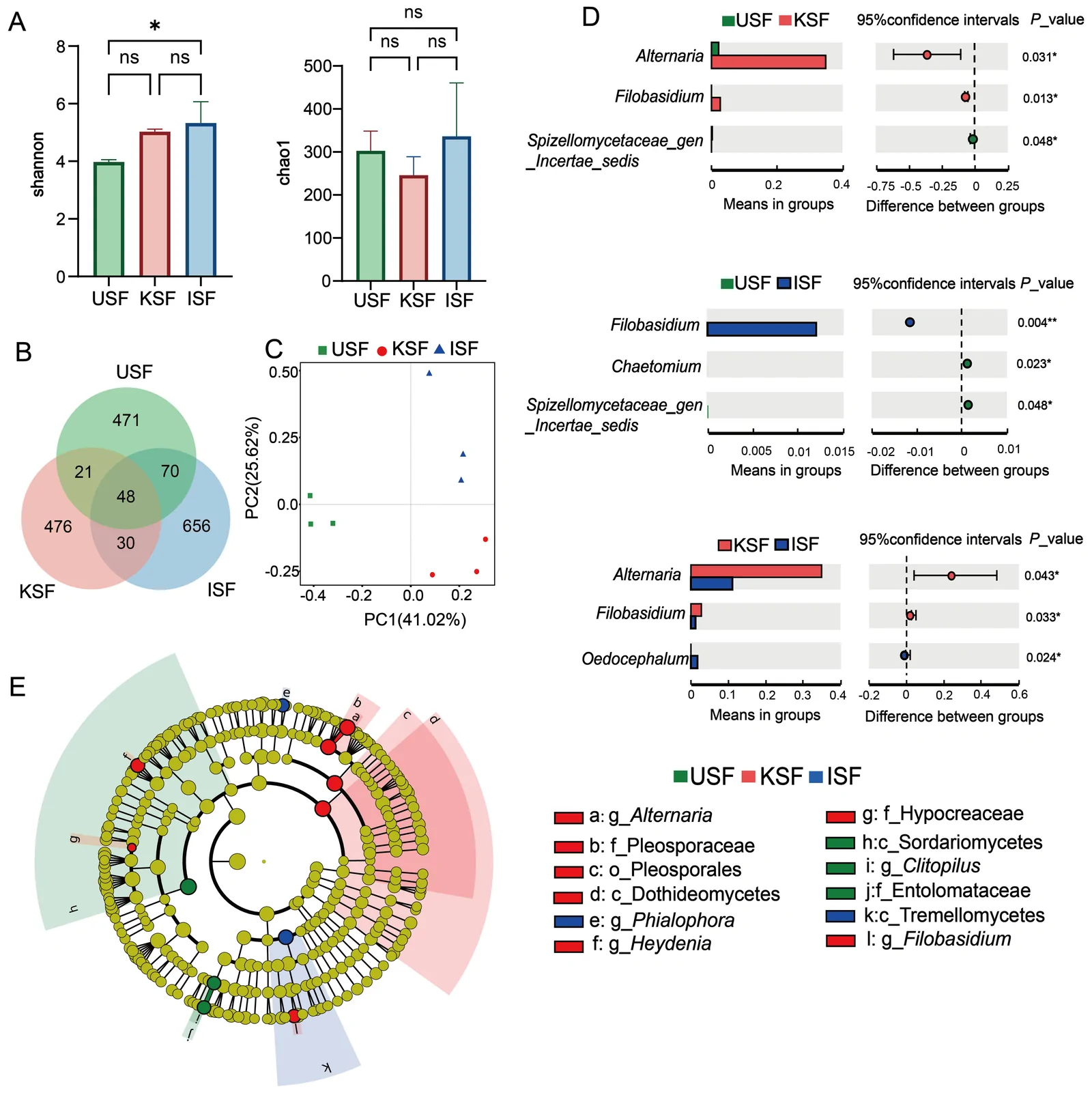
Fungal diversity and species differences in the different habitats. *USF*, Urumqi soil fungi; *KSF*, Karamay soil fungi; *ISF*, Ili soil fungi. *p < 0.05; **p < 0.01; ***p < 0.001. **(A)** Alpha diversity indices among USF, KSF, and ISF groups (ns: not significant). **(B)** Venn diagram showing the shared and unique OTUs/ASVs among the three groups. **(C)** PCoA plot based on fungal community composition displaying the separation of the groups. **(D)** Differential abundance analysis of fungal genera between groups with 95% confidence intervals and P-values. **(E)** Circos plot illustrating the fungal community composition and interactions across different taxonomic levels and habitats.

Urumqi had the highest bacterial diversity and richness in the rhizosphere soil of *C. spinosa*, and the dominant bacterial group, *Rubrobacter*, has been reported in related studies to participate in the mineralization of soil organic matter, which can promote the activation and cycling of soil nutrients, help plants absorb nutrients more efficiently, and further promote the accumulation of active components in fruits. Soil metabolites enriched in lipid and amino acid metabolism in Urumqi are at significantly higher levels than those in Karamay. These small-molecule metabolites can not only directly provide precursors for the synthesis of medicinal active ingredients of *C. spinosa* but also interact with the dominant microbial community in the soil to jointly maintain a good rhizosphere nutrient environment, ultimately promoting the formation of high-quality fruits in Urumqi.

### 2.5 Correlation between the quality differences of *C. spinosa* and soil factors

Significant differences have been previously found in the effective components of *C. spinosa* across different habitats. To elucidate this phenomenon, a correlation analysis was conducted by integrating the soil metabolites and soil microorganisms. Seven fruit active ingredients (resveratrol, nodakenin, phloretin, eriodictyol, luteolin, trifolin, and kaempferol) were significantly positively correlated with eight soil metabolites (glucobrassicin, 2-ketoadipic acid, l-serine, LPC16:1, LPC18:3, LPC18:2, l-palmitoylcarnitine, and succinic acid) (*p* < 0.05). At the same time, four medicinal components—resveratrol, ferulic acid, gentiopicrin, and verbenalin—were significantly positively correlated with seven soil metabolites (palmitic acid, l-aspartic acid, citric acid, alpha-ketoglutaric acid, l-glutamate, l-arginine, and 2-isopropylmalic acid) (*p* < 0.05). The remaining five active ingredients (diosmetin, 3,4-dihydroxybenzaldehyde, butein, phloridzin, and hesperetin) were significantly positively correlated with glucobrassicin, 2-ketoadipic acid, and l-serine (*p* < 0.05). In addition, pristimerin was significantly positively correlated with glucobrassicin and 2-ketoadipic acid, but significantly negatively correlated with l-arginine (**Figure 5**).

**Figure 5:**
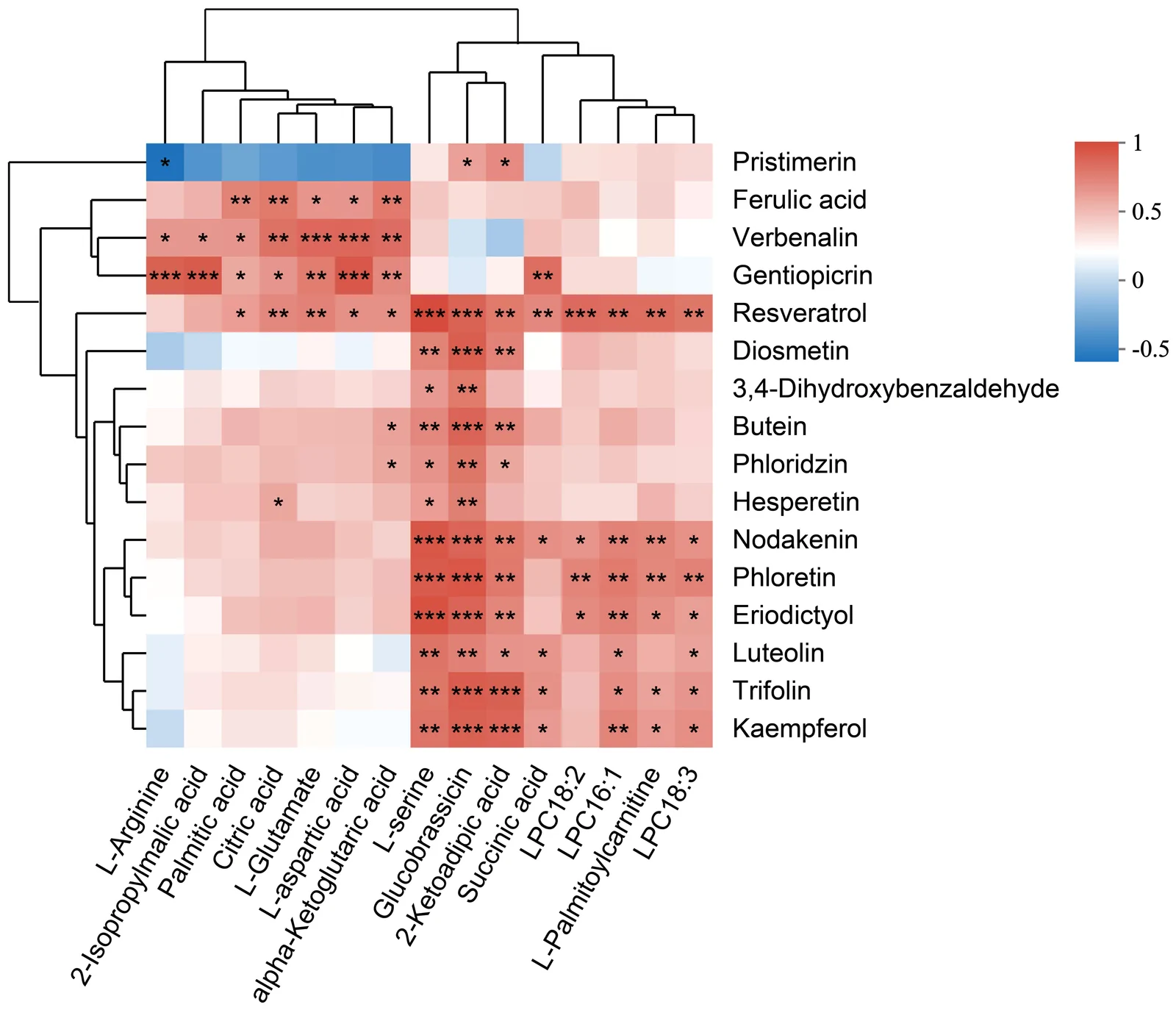
Impact of soil metabolites on the fruit quality of *Capparis spinosa*.

Differential microorganisms in various habitats are also closely associated with the quality of *C. spinosa* (**Figure 6**). Flavonoids and phenolic components, including luteolin, trifolin, kaempferol, phloretin, diosmetin, 3,4-dihydroxybenzaldehyde, resveratrol, eriodictyol, nodakenin, phloridzin, butein, and hesperetin, were significantly positively correlated with *Gaiella*, *Spizellomycetaceae_gen_incertae_sedis*, *Rubrobacter*, and *Clitopilus* and significantly negatively correlated with *Blastococcus*, *Nocardioides*, *Kocuria*, *Alternaria*, and *Filobasidium*. Pristimerin exhibited a negative correlation with *Blastococcus* and a positive correlation with *Clitopilus* (*p* < 0.05). Ferulic acid, gentiopicrin, and verbenalin were significantly negatively correlated with *Pontibacter* and *Heydenia* and significantly positively correlated with *Candidatus_Nitrocosmicus*, *Sphingomonas*, *Oedocephalum*, and *Rubellimicrobium* (*p* < 0.05).

**Figure 6:**
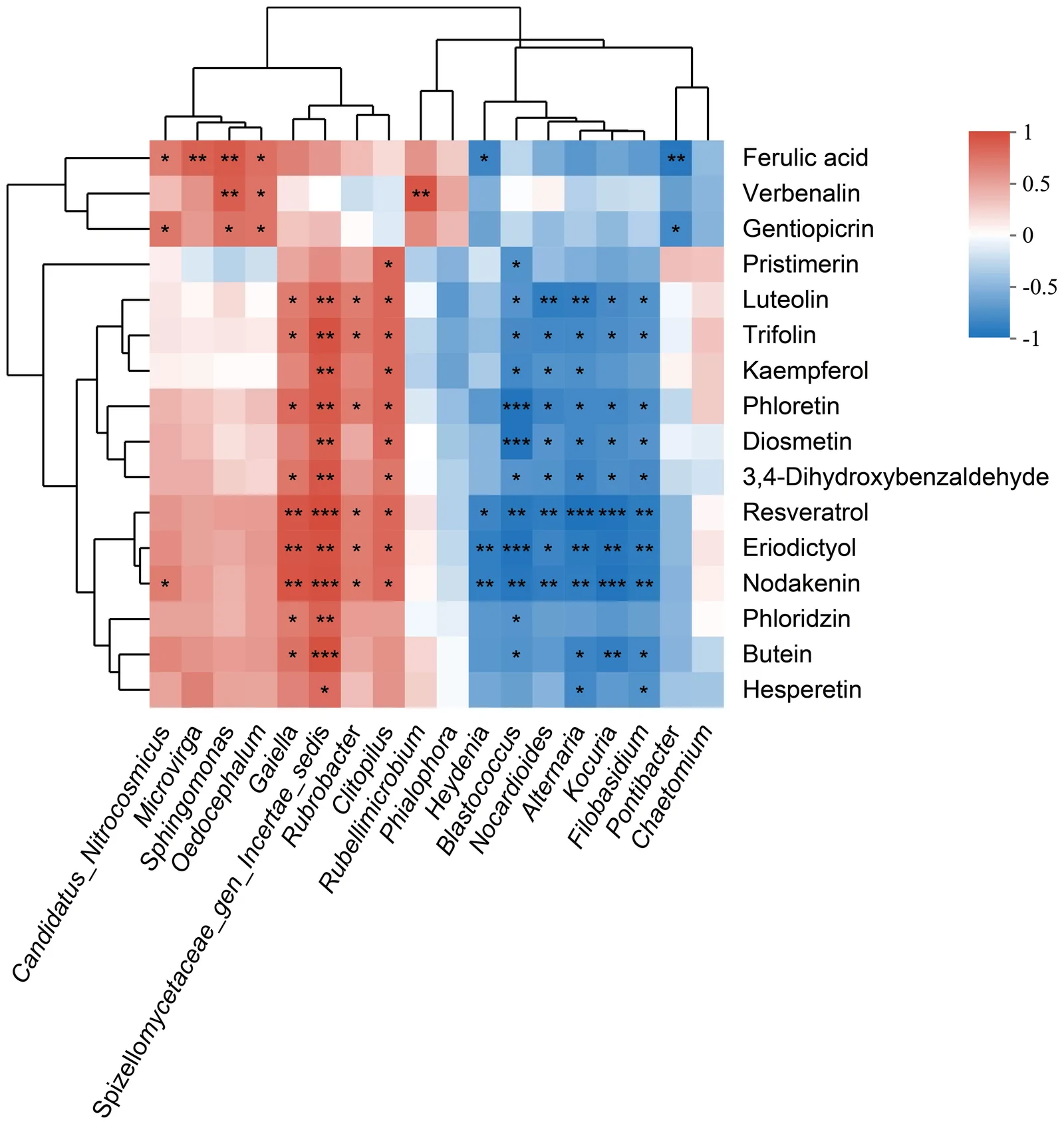
Influence of differential microorganisms on the quality of *Capparis spinosa*. *p < 0.05; **p < 0.01; ***p < 0.001.

With regard to interpretation, it should be noted that the correlation analyses reported above were performed on a pre-selected subset of metabolites and microbial taxa (the top differential fruit and soil metabolites and the top differential microbial genera, selected by fold change (FC) ranking; see *Section 4.4*) rather than the full metabolome or microbiome. These associations should therefore be interpreted as potential, hypothesis-generating relationships rather than as evidence of causal regulation or as a comprehensive picture of soil–microbe–fruit interactions.

## 3 Materials and methods

### 3.1 Chemicals and plant materials

Among the reagents used in the metabolome analysis section, methanol and formic acid (LC-MS grade) were from Thermo Fisher Scientific (Waltham, MA, USA). The high-purity characteristics of these chemicals ensure the accuracy and reproducibility of the experimental analysis. Water (LC-MS grade) was provided by Merck (Darmstadt, Germany). LC-MS grade reagents have strictly controlled impurity levels, which can effectively reduce background interference and ion suppression in non-targeted metabolomics detection based on LC-MS/MS. This ensures high sensitivity and mass accuracy for the identification and quantification of the 16 putative anti-inflammatory-associated metabolites in *C. spinosa* L. fruits, avoiding false-positive signals or quantitative deviations caused by reagent impurities, and guarantees the reliability and reproducibility of the qualitative and quantitative results of the active components.

Samples were collected in late August 2023. *C. spinosa* fruits were collected in Urumqi (87°43'53.9" E, 43°51'20.7" N), Karamay (85°5'11.8" E, 45°36'23.6" N), and Ili (82°8'32.8" E, 43°37'0.4" N). In each region, six representative quadrats were determined, and 8–10 mature fruits of *C. spinosa* were collected from each quadrat. Survey plots were established at random in accordance with the distribution characteristics of the prickly ash populations. The area of each plot was set to correspond to the area covered by a single population, with each plot generally covering no less than 1,000 metre^2^. These fruits were denoted as UF (Urumqi fruits), KF (Karamay fruits), and IF (Ili fruits). Simultaneously, rhizosphere soil at 0- to 20-cm depth of *C. spinosa* was collected using the five-point sampling method and the principle of random multi-point mixing within each quadrat. After the soil samples were passed through a 2-mm sterilized sieve, the samples were labeled as US (Urumqi soil), KS (Karamay soil), and IS (Ili soil) and then loaded into sealed bags. The fruit and soil samples collected from each region were used for metabolome analysis with six replicates. The six biological replicates correspond to the six independent quadrats described in the sampling design. In this study, six biological replicates were set following the standard design convention for metabolome and microbial diversity analysis: this number of replicates can effectively capture the natural variation of samples within each habitat, reduce the impact of random individual variations on the experimental results, and improve the statistical power for detecting significant differences between groups, which effectively guarantees the reliability and robustness of the subsequent analysis and conclusions. Moreover, another portion of the soil samples was utilized to identify variations in the soil microorganisms. The soil bacteria from the three habitats were represented as USB (Urumqi soil bacteria), KSB (Karamay soil bacteria), and ISB (Ili soil bacteria), while the soil fungi were represented as USF (Urumqi soil fungi), KSF (Karamay soil fungi), and ISF (Ili soil fungi). All samples were stored at low temperatures.

### 3.2 Sample preparation and LC-MS/MS analysis conditions

For metabolite extraction, 100 mg of tissue samples milled with liquid nitrogen was transferred into EP tubes. Subsequently, 500 μl of an 80% methanol aqueous solution was added, and the mixture was vortexed, shaken, and left to stand on an ice bath for 5 min. The samples were then centrifuged at 15,000 × *g* for 20 min at 4°C. After centrifugation, the supernatant was collected and diluted with mass spectrometry-grade water to achieve a 53% methanol concentration. Following a second centrifugation step at 15,000 × *g* for 20 min at 4°C, the resulting supernatant was collected and subjected to LC-MS detection and analysis (Dunn et al., 2011).

For LC-MS/MS untargeted metabolomics analysis, the metabolites were separated using a Hypersil Gold C18 chromatographic column on the UHPLC-Q Exactive HF-X system. The column was maintained at 40°C with a flow rate of 0.2 ml/min, and the total chromatographic elution time was 17 min. The mobile phase consisted of 0.1% formic acid (mobile phase A) and methanol (mobile phase B). Mass spectrometry data were collected in both positive and negative ionization modes, with a scan range of 100–1,500 *m*/*z*. After data acquisition, raw LC-MS files were imported into Compound Discoverer 3.3 (Thermo Fisher Scientific, Waltham, MA, USA) for preprocessing, including baseline correction, peak alignment, peak picking, and signal integration. Preprocessed features were filtered to remove low-quality noise and non-biological interference using the following criteria: 1) features with retention time <0.5 min were excluded to eliminate non-retained solvent contaminants; 2) features with *m*/*z* outside the range of 80–1,600 were removed to match the actual scanning range of the mass spectrometer; and 3) features with maximum signal intensity across all samples less than 1,000 were filtered out to remove background instrument noise. After filtering, the metabolites were matched against the CD 3.3 built-in database to retrieve the peak area, the mass-to-charge ratio (*m*/*z*), and other parameters for quantification. KEGG and LIPID MAPS (LIPID Metabolites and Pathways Strategy) were used for the annotation and classification of the fruit and soil metabolites. All subsequent statistical analyses were performed in R. MetaX software (Wen et al., 2017) was employed for the PLS-DA of the metabolic data from the different habitats. Differential metabolites were identified based on the criteria of variable importance in the projection (VIP) > 1, *p* < 0.05, and FC ≥ 2 or FC ≤ 0.5, with *p* < 0.05 considered statistically significant. KEGG database (https://www.genome.jp/kegg/pathway.html) was used for pathway enrichment analysis, and pathways with *p* < 0.05 were defined as significantly enriched.

For untargeted metabolomics identification, metabolite annotation was performed by matching accurate *m*/*z* and MS/MS fragmentation against spectral libraries. The resulting annotations are putative identifications (Metabolomics Standards Initiative Level 2–3) that were not confirmed with authentic reference standards and should therefore be interpreted with caution. The signal intensities used in this study reflect relative (semi-quantitative) responses rather than absolute concentrations. Therefore, all between-habitat comparisons refer to relative abundance rather than absolute content. As the annotation of untargeted features is putative and many plant metabolites are incompletely represented in pathway databases, the KEGG enrichment results are only interpreted at the level of broad metabolic categories.

### 3.3 Microbial sequencing and data analysis methods

DNA extraction was conducted following the instructions in the FastDNA^®^ SPIN Kit for Soil (MP Biomedicals, Santa Ana, CA, USA). Using the universal primers 338F (5'-ACTCCTACGGGGAGGCAGCAG-3') and 806R (5'-GGACTACHVGGGTWTCTAAT-3'), the V3–V4 region of the 16S rRNA gene was amplified (Xu et al., 2016). For amplification of the ITS region, the primers ITS1F (5'-CTTGGTCATTTAGAGGAAGTAA-3') (Xia et al., 2024) and ITS2R (5'-GCTGCGTTCT-TCATCGATGC-3') (He et al., 2017) were employed. PCR amplification was carried out using an ABI GeneAmp^®^ 9700 PCR instrument (Foster City, CA, USA), and the purified PCR products were sequenced on the Illumina MiSeq PE250 platform (San Diego, CA, USA).

After quality control, filtering, and splicing with fastp (v0.19.6) and FLASH (v1.2.11) software, valid sequences were obtained. OTUs were clustered at a 97% similarity level using UPARSE (v7.1) software. The representative sequences of the OTUs were annotated for species classification via the Bayesian algorithm implemented in the RDP classifier (v11.5). A 97% similarity threshold for OTU clustering was adopted to remain consistent with the reference-based classification pipeline (UPARSE/RDP classifier) used throughout this study and to allow direct comparison with the substantial body of regional soil microbiome literature that also relies on OTU-based clustering. We acknowledge that amplicon sequence variant (ASV)-based methods (e.g., DADA2), which resolve single-nucleotide differences without a fixed similarity threshold and offer finer taxonomic resolution, represent a valuable methodological refinement and a direction for future reanalysis of this dataset. The mothur software was used to calculate the alpha diversity indices for each sample, and Student’s *t*-tests were performed to generate dilution curves and box plots. Given the sample size (*n* = 6 biological replicates per habitat) and the approximately symmetric distribution of the diversity indices after replicate averaging, Student’s *t*-test was considered an appropriate parametric approach for pairwise comparisons of the alpha diversity between habitats, consistent with common practice in comparative soil microbiome studies (e.g., as implemented in mothur and STAMP). R software (v4.2.3) was utilized to visualize the species composition. Beta diversity was calculated using QIIME (v1.9.1) to construct the PCoA plots based on Bray–Curtis dissimilarity, a compositional data-appropriate distance metric for microbial community structure. Differential species were identified through *t*-tests and LEfSe. Specifically, the genus-level pairwise abundance differences were assessed using *t*-tests as a parametric screen and were cross-validated with the non-parametric Kruskal–Wallis test embedded in the LEfSe framework, which does not assume normality and is more robust to the compositional, non-normally distributed nature of the relative abundance microbiome data. The concordance of the differential taxa identified by both approaches supports the robustness of the reported results.

### 3.4 Determination of key factors affecting differential metabolites

The top 15 metabolites with significant differences in the fruits and soil of *C. spinosa* from the different habitats were selected to analyze their correlations. Simultaneously, the correlations between the top 16 differential metabolites in the fruits and the top 10 differential microbial species with significant differences in the soil were analyzed. The corrplot software package was employed for the correlation analysis, and a significant relationship was considered when *p* < 0.05. A heatmap was used to visualize the results of the correlation analysis.

For the metabolites, we first performed univariate analysis and pre-screened differential metabolites that satisfied the thresholds of *p* < 0.05 (from the univariate test), VIP > 1, and FC ≥ 2 or ≤0.5. After sorting the screened differential metabolites by the absolute value of log_2_FC in descending order, we selected the top 15 differential soil metabolites and the top 16 differential fruit metabolites for subsequent correlation analysis. It should be noted that the retained 16 fruit metabolites also matched the 16 reported anti-inflammatory active ingredients of *C. spinosa* from public medicinal databases, which is consistent with our study design. For the soil microbial species, we first used the thresholds of *p* < 0.05 (from the Kruskal–Wallis test in the univariate analysis) and linear discriminant analysis (LDA) score >2 to identify significant differential genera between the different habitats via LEfSe; sorted these differential genera by the LDA score, which represents the effect size of inter-habitat differences, in descending order; and finally selected the top 10 differential genera for correlation analysis with the fruit metabolites.

## 4 Discussion

### 4.1 The active components of *C. spinosa* are significantly different in different habitats

The therapeutic value of medicinal plants is primarily evaluated by the active ingredients with pharmacological functions within the plant. The 16 putative anti-inflammatory-associated metabolite effects in *C. spinosa* fruits selected in this study are composed of flavonoids, terpenoids, and phenylpropanoids, with flavonoids being the predominant compounds. This composition is similar to that of many traditional Chinese medicines (TCMs), including *Cinnamomum longepaniculatum* (Xie et al., 2025), *Dendrobium officinale* (Hua et al., 2019), and *Codonopsis convolvulacea* Kurz (Zong et al., 2025; Sun et al., 2025). Flavonoids, as crucial active components of medicinal plants (Weston and Mathesius, 2013), exhibit antioxidant activity (Fotios et al., 2023), UV protection, and antibacterial and antiviral properties (Muhammad et al., 2018). They function as the primary bioactive constituents in many medicinal plants and are crucial for pharmaceutical development. In addition, terpenoids are beneficial for enhancing human health (Ayu et al., 2022). For instance, artemisinin is a well-known antimalarial drug (Marchev et al., 2021), and ginkgolide, a natural platelet activation antagonist, possesses neuroprotective and repair functions (Osei et al., 2025). Phenylpropanoids, which constitute the largest group of active components in higher plants, have antioxidant, UV-resistant, anticancer, antiviral, anti-inflammatory, wound healing-promoting, and antibacterial properties (Zhu et al., 2023), indicating high medicinal potential.

However, the effective components of medicinal plants are closely intertwined with their habitat conditions. Metabolome analysis revealed that the relative levels of the active ingredients in *C. spinosa* fruits differed significantly among habitats. Comparative analysis showed that majority of the flavonoid class active ingredients had higher relative levels in the Urumqi samples, while terpenoids (gentiopicrin and verbenalin), hesperetin, and ferulic acid were more abundant in Ili fruits. This suggests that the *C. spinosa* fruits from Urumqi showed the highest relative levels of these components, with relatively lower levels in Ili and Karamay. These results indicate that the medicinal plant quality varies with habitat. However, as an observational, non-manipulative comparison, this study cannot establish habitat as a direct causal driver of quality. This finding aligns with research conclusions regarding *Eucommia ulmoides* (Hua et al., 2020), Daohuaxiang rice (Kabir et al., 2024), *Artemisia rupestris* L (Jiang et al., 2025), black tea (Zhang et al., 2025), and *Chrysanthemum indicum* (Zou et al., 2022) from diverse habitats. Secondary metabolite production is influenced by environmental factors, and majority of plants adjust the type and quantity of these metabolites according to their growth environment (Juan'e et al., 2006; Valls et al., 2006). Consequently, a new interdisciplinary field, the Quality Ecology of Chinese Medicines (QECM) (Ji et al., 2024), has emerged, catalyzing the refinement of the current standard system of TCMs and driving the internationalization of the TCM industry forward.

### 4.2 The soil metabolites of *C. spinosa* differ significantly among different habitats

Soil metabolites, an important indicator reflecting soil nutrient status (Yang X et al., 2021), can reveal the formation and transformation of soil substances and provide a crucial basis for the evaluation of soil environmental conditions. They encompass not only the root exudates from plants but also the microbial metabolites and decomposition products of plants, microorganisms, and soil organic matter (Cheng et al., 2018). Root exudates, in particular, refer to the various organic and inorganic substances released by plant roots into the surrounding soil environment, including sugars, amino acids, organic acids, and active components (Badri and Vivanco, 2009). During degradation, these substances form elements that plants can absorb and utilize, participating in diverse biochemical reactions.

As a key component of soil organic matter, fatty acids release carbon and energy through degradation and metabolism, which soil microorganisms can use for nutrient transformation and which, in general, can also supply carbon that supports plant growth and secondary metabolite biosynthesis. Organic phosphorus mineralization may likewise increase the bioavailability of soil phosphorus, a nutrient generally important for energy metabolism and secondary metabolite synthesis in plants, and the amino acids released from soil organic matter may contribute to the nitrogen supply that supports protein and nitrogen-containing metabolite synthesis (Ding et al., 2020; Moreira and Moraes, 2017). These are general soil nutrient functions reported in the literature. The present correlational dataset did not directly examine whether they regulate the accumulation of active ingredients in *C. spinosa*. Therefore, this proposed link should be regarded as a hypothesis rather than a demonstrated mechanism.

Given the potential relevance of these nutrient cycling pathways to *C. spinosa* quality, this study focused on the lipid metabolism, amino acid metabolism, and glycerophospholipid metabolism pathways among the numerous soil metabolite processes. The comparison of 15 differential soil metabolites revealed significant variations in the soil metabolite levels across different habitats. The results further suggest that the supply of carbon, nitrogen, and phosphorus (nutrients critical for *C. spinosa* quality formation) is more sufficient in Urumqi and Ili, supporting higher accumulation of active components in harvested materials, while in Karamay, the contents of soil metabolites related to these nutrient cycling processes are generally low. This pattern is consistent with, but does not by itself demonstrate, insufficient nutrient supply constraining active ingredient accumulation in the material from Karamay; however, targeted experiments would be needed to confirm this link. Such variations can be attributed to the unique geographical and climatic conditions of the three habitats. For instance, the organic matter content typically increases with altitude (Hua et al., 2020). In addition, varying geographical and climatic conditions alter the soil microbial metabolic patterns, influencing the distribution of soil metabolites (Chen D et al., 2021). These factors may have contributed to the observed quality differences in *C. spinosa* across the different habitats, although the relative contribution of each factor was not directly assessed here.

### 4.3 Different habitats have a significant impact on microbial diversity and community structure

The environmental conditions across different geographical regions, including climate, soil types, and vegetation, impose varying selection pressures on microbial communities. These pressures drive adaptive differentiation, shaping microbial diversity and community composition (Yang et al., 2021), with geographical distance identified as the primary driving factor (Devin et al., 2016). These findings align with previous research on soil microorganisms associated with medicinal plants from diverse habitats, such as *Codonopsis pilosula* (Meng et al., 2023), *Cynanchum bungei* Decne (Pawlik et al., 2020), ginger (Li and Li, 2026), and *E. ulmoides* Oliv (Zhang et al., 2025). After long-term natural selection and adaptation, medicinal plants from different regions assemble specific rhizosphere microbial communities that support their growth and quality formation.

In this study, the dominant bacterial phyla identified were Actinobacteria, Proteobacteria, Chloroflexia, and Bacteroidetes, which is consistent with the dominant bacterial communities observed in the majority of other medicinal plant systems (Gu et al., 2021). As two major soil bacterial groups, Actinobacteria and Proteobacteria play crucial roles in degrading insoluble organic matter and driving soil nutrient cycling (Gustavo, 2022; Yuan et al., 2025). In addition, Actinobacteria can produce bioactive molecules such as antibiotics and enzymes, which contribute to the inhibition of plant pathogens and the maintenance of rhizosphere health (Sun et al., 2025). Chloroflexia, commonly detected in saline–alkali paddy soils (Liu et al., 2022), participates in organic matter degradation, nitrogen removal, and the enhancement of plant abiotic stress resistance (Bovio et al., 2023). Bacteroidetes contributes to soil nutrient transformation and the improvement of soil fertility, creating favorable growth conditions for plants (Anna et al., 2023).

At the genus level, we observed clear differentiation of the dominant bacteria across the three study habitats, which parallels previous findings that habitat-specific dominant bacterial genera drive quality variation in the medicinal plant *Cynomorium songaricum* (Yang Y et al., 2024). In this study, *Rubrobacter* was the dominant bacterial genus in US: previous research has reported that *Rubrobacter* abundance increases under drought conditions (the typical climate of Urumqi), and this genus has been reported to participate in the decomposition of recalcitrant organic matter, which could plausibly support soil nutrient availability and, in turn, active component accumulation in *C. spinosa* (Duan et al., 2022).; This pathway was not directly examined in the present study. In KS, the dominant bacterial genera were *Salinimicrobium*, *Halomonas*, *Kocuria*, and *Pontibacter*. Among them, *Kocuria* has been reported to promote plant hormone secretion, increase soil nutrient bioavailability, and enhance plant salt–alkali tolerance in other systems and generally shows higher activity in the rhizosphere environment (Hassanzadeh et al., 2024; Li et al., 2023). *Halomonas* and *Salinimicrobium* are highly salt-tolerant taxa that help alleviate salt stress in plants growing in Karamay’s saline soils, supporting healthy plant growth (Liu et al., 2026; Sun et al., 2020). In IS, the dominant genus, *Sphingomonas*, plays a role in organic pollutant degradation, plant hormone synthesis, nitrogen fixation, phosphate solubilization, and heavy metal tolerance, functions that have been reported to benefit plant growth and quality formation in other species (Jiang et al., 2016; Asaf et al., 2020) and may plausibly contribute similarly in *C. spinosa*.

Consistent with the bacterial results, significant differences were also detected in the soil fungal diversity and community composition across different habitats, which aligns with previous observations in other medicinal plants including *Paris polyphylla* and *Glycyrrhiza inflata* (Pawlik et al., 2020; Yuan et al., 2025). Soil fungal communities assemble unique structures to adapt to local habitat conditions. At the phylum level, Ascomycota and Basidiomycota serve as the primary decomposers in soil, driving soil nutrient cycling to support plant growth (Manici et al., 2024). At the genus level, clear habitat-specific distinctions were observed in the dominant fungal taxa. With the exception of a small number of pathogenic taxa, majority of the soil-associated fungi provide positive benefits for plant growth. For example, comparative analyses of endophytic fungal communities in healthy tea leaves and leaves affected by tea wheel spot disease revealed that beneficial genera, including *Mortierella*, *Aspergillus*, *Plectosphaerella*, *Lecythophora*, *Botryotrichum*, *Penicillium*, *Gibberella*, *Chaetomium*, *Lulwoana*, and *Verticillium*, were significantly more abundant in healthy leaves, indicating their potential roles in biological disease control (Sidhu et al., 2024). In our study, the dominant fungal genus *Alternaria* showed habitat-specific differences in abundance: previous work has reported that *Alternaria* can promote plant tolerance to abiotic stress and participate in the transformation of soil organic matter, and its varied abundance across habitats may plausibly contribute to the growth and quality differences of *C. spinosa* across regions. However, this link was not directly assessed here.

### 4.4 The quality differences of *C. spinosa* in different habitats are highly correlated with soil factors

The soil environment is intricately connected with plant growth and development. Studies have revealed that the soil metabolites and microorganisms in diverse habitats can significantly impact the quality of *C. spinosa*. This is because soil metabolites supply essential nutrients to plants during degradation, facilitating the accumulation of the medicinal components in *C. spinosa*. At the same time, soil microorganisms, as key drivers of geochemical cycling and nutrient flow, modulate soil metabolism and plant hormone levels, or assist plants in the absorption of more nutrients, thereby promoting the growth and accumulation of active ingredients (Peng et al., 2020). According to relevant research, the soil microorganisms of *Salvia miltiorrhiza* (He et al., 2023), *Fritillaria taipaiensis* (Mu et al., 2019), and *Cinnamomum migao* (Che et al., 2026) have been reported to be associated with the synthesis of plant active ingredients. A study also collected mature fruits of medicinal plants from various locations, conducted metabolome detection, determined the active components, and performed correlation analysis with rhizosphere soil microorganisms. It was found that fungal groups (*Agaricus*, *Fusarium*, *Phialophora*, *Cryptococcus*, *Trichosporon*, and *Archaeorhizomyces*) were significantly positively correlated with three medicinal ingredients (i.e., 1,8-cineole, sabinene, and *O*-terpineol) (Song et al., 2025) (**Table 1**).

**Table 1:** The correlation between soil metabolites/microorganisms and fruit quality.

Soil metabolites	Resveratrol	Phloretin	Pristimerin	Trifolin	Kaempferol	Eriodictyol	Luteolin	Butein	Phloridzin	Diosmetin	Nodakenin	Hesperetin	Verbenalin	Gentiopicrin	3,4-Dihydroxybenzaldehyde	Ferulic acid
Glucobrassicin	0.419	0.420	0.025	0.467	0.452	0.534*	0.520*	0.696**	0.549*	0.872**	0.596**	-0.032	-0.053	0.144	0.602**	-0.070
L-serine	0.467	0.453	0.313	0.677**	0.671**	0.592**	0.640**	0.339	0.188	0.537*	0.730**	0.048	-0.057	-0.033	0.407	-0.067
L-Palmitoylcarnitine	-0.120	-0.118	0.334	0.301	0.340	0.588*	0.271	-0.006	-0.134	-0.043	0.259	-0.013	-0.103	-0.094	-0.089	-0.034
LPC 16:1	0.205	0.206	0.160	0.300	0.300	0.941**	0.312	0.327	0.131	0.391	0.383	-0.082	-0.052	-0.109	0.220	-0.058
alpha-Ketoglutaric acid	-0.190	-0.181	-0.287	-0.266	-0.262	0.323	-0.274	0.318	0.280	-0.120	-0.116	0.504*	0.342	0.267	0.197	0.447
LPC 18:3	0.150	0.141	0.399	0.631**	0.661**	0.480*	0.532*	0.059	-0.001	0.235	0.521*	-0.035	-0.154	-0.121	0.149	-0.068
L-aspartic Acid	-0.130	-0.130	-0.277	-0.221	-0.223	-0.150	-0.176	0.005	0.029	-0.190	-0.164	0.213	0.902**	0.744**	-0.100	0.858**
2-Ketoadipic acid	0.338	0.324	0.357	0.565*	0.560*	0.193	0.527*	0.244	0.103	0.529*	0.650**	-0.110	-0.193	0.265	0.271	-0.113
LPC 18:2	0.083	0.077	0.021	0.215	0.216	0.858**	0.163	0.182	0.080	0.380	0.283	-0.085	0.086	-0.058	0.224	0.073
L-Glutamate	0.021	0.016	-0.225	-0.029	-0.036	-0.002	0.008	0.104	0.076	-0.029	0.069	0.262	0.866**	0.765**	0.006	0.680**
Citric acid	-0.109	-0.107	-0.260	-0.166	-0.161	-0.114	-0.167	-0.016	0.017	-0.208	-0.160	-0.005	0.823**	0.643**	-0.095	0.764**
Palmitic acid	0.028	0.024	-0.063	0.172	0.183	0.404	0.182	0.226	0.085	0.035	0.288	0.258	0.395	0.503*	0.098	0.546*
L-Asparagine	-0.104	-0.106	-0.266	-0.194	-0.198	-0.116	-0.148	0.036	0.059	-0.175	-0.124	0.342	0.882**	0.729**	-0.067	0.743**
Succinic acid	0.435	0.429	0.192	0.598**	0.601**	0.110	0.627**	0.366	0.242	0.236	0.607**	0.200	0.279	0.547*	0.238	0.322
L-Arginine	-0.142	-0.141	-0.325	-0.243	-0.245	-0.218	-0.197	0.021	0.084	-0.207	-0.187	0.281	0.863**	0.776**	-0.067	0.790**

* indicates P < 0.05, significant correlation; ** indicates P < 0.01, highly significant correlation. Data were analyzed using paired-sample Pearson correlation analysis with samples from 3 sampling sites (w/k/y) × 6 biological replicates (n=18).

Our findings indicate that certain microbial groups exhibit significant correlations with plant metabolites. Among them, *Rubrobacter*, *Gaiella*, *Clitopilus*, and *Spizellomycetaceae*_*gen_Incertae_sedis* are the dominant microorganisms in the soil of Urumqi and are closely associated with more than half of the active components, such as phenols and flavonoids, in *C. spinosa*. Research has reported that *Rubrobacter*, which belongs to the phylum Actinobacteria, is resistant to drought and radiation environments (Basile et al., 2025). By decomposing soil organic matter, it releases precursor substances for valine, leucine, and isoleucine, which has been linked to the synthesis of these amino acids (Huang et al., 2024) and could plausibly contribute to the accumulation of active components in plants. *Gaiella*, also belonging to the phylum Actinobacteria, is involved in soil organic carbon decomposition and nutrient cycling (Miao et al., 2020), thereby enhancing soil fertility and productivity. *Clitopilus*, a member of the phylum Basidiomycota, can produce diterpene antibiotics (Zhao et al., 2021) to protect plants from pathogens and form symbiotic relationships with plants to facilitate nitrogen absorption from the soil (Yang et al., 2024), which has been linked to plant growth and quality in other systems. These reported effects, drawn from other plant systems, may be relevant to agricultural soil management and to the flavonoid and phenol accumulation in *C. spinosa*; however, this was not directly examined here. In contrast, *Alternaria* and *Filobasidium* are pathogenic bacteria that cause plant wilting, leaf spots, and rot (Sadhana et al., 2023). *Alternaria* can produce several toxic active components, known as *Alternaria* mycotoxins, under specific conditions. These mycotoxins are cytotoxic and significantly reduce the yield and quality of important crops, vegetables, and fruits (Chen A et al., 2021), imposing negative impacts on agricultural economies. Moreover, studies have reported that *Alternaria* and *Filobasidium* associated with the roots of *E. ulmoides* are negatively correlated with the levels of effective components such as aucubin, geniposidic acid, and pinoresinol diglucoside (Norese et al., 2025). At the genus level, *Clitopilus* is a beneficial biomarker of US, while *Alternaria* and *Filobasidium* are pathogenic biomarkers of KS. Moreover, the soil metabolites with positive correlations showed the highest relative levels in the Urumqi samples and lower levels in KS. This co-occurrence is consistent with, but does not establish, a role for these taxa in the higher relative levels of the active components observed in Urumqi *C. spinosa* compared with Karamay. Regarding *Kocuria*, prior studies have reported generally positive effects on the accumulation of active components in other medicinal plants. For example, *Kocuria rhizophila*, a plant growth-promoting bacterium (PGPB), can produce indole acetic acid, solubilize phosphorus, and tolerate salt and drought stress. Multi-omics studies on the PGP activity of *K. rhizophila* in tomatoes have shown that it can regulate molecular pathways related to plant growth, encompassing amino acid metabolism, cell wall organization, lipid metabolism, and secondary metabolism (Özcan and Efe, 2024). However, these results are in contrast with those of this study, indicating that the influence of *Kocuria* on active components is selective.

For terpenoids, the primary influencing factors include amino acid metabolites such as citric acid, 2-isopropylmalic acid, l-aspartic acid, l-glutamate, 2-ketoadipic acid, and soil microorganisms such as *Candidatus_Nitrocosmicus*, *Sphingomonas*, *Oedocephalum*, *Microvirga*, and *Rubellimicrobium*. We observed significant positive correlations between the abundance of certain microbes, such as *Sphingomonas* and *Microvirga*, and the levels of key terpenoid components. Among these, amino acid metabolites regulated by microorganisms can indirectly enrich soil nitrogen. Research on other systems has shown that *Sphingomonas* species can play a positive role in plant growth promotion by producing plant hormones [e.g., gibberellin, salicylic acid (SA), indole-3-acetic acid (IAA), zeatin, and abscisic acid], which stimulate plant germination and growth (Asaf et al., 2020). They also facilitate nutrient cycling of nitrogen, carbon, sulfur, and iron (Wang et al., 2024), potentially enhancing plants’ nutrient absorption and utilization from the soil. In addition, *Sphingomonas* (Zhang et al., 2020) has shown a positive correlation with emodin, a major bioactive compound in the roots of *Polygonum cuspidatum*. Relevant studies have demonstrated that the luteolin in *Scutellaria baicalensis* (Dong et al., 2024) is extremely significantly positively correlated with *Microvirga* and *Sphingomonas*, consistent with the findings of this study, suggesting that these are beneficial microorganisms for active component synthesis. Conversely, *Kocuria*, *Nocardioides*, *Blastococcus*, *Alternaria*, and *Filobasidium*, as dominant microorganisms in KS, are negatively correlated with soil metabolites. Possible explanations proposed in the literature for such negative correlations include resource competition, metabolic inhibition, oxidative stress, and depletion of defense resources, which could have plausibly contributed to the lower relative levels of active components observed in Karamay *C. spinosa* fruits. However, this mechanism was not directly assessed in the present study. These correlational results indicate that the medicinal components of *C. spinosa* are associated with soil factors. Favorable soil nutrient conditions and specific microbial taxa may contribute to the accumulation of medicinal components; however, the causal regulation of this accumulation was not established in this study.

Taken together, these correlational findings advance our understanding of how local habitat conditions are related to rhizosphere microbiome assembly and to plant quality in *C. spinosa*, but also deliver actionable insights for the sustainable cultivation and downstream pharmacological development of this medicinal species. From a cultivation perspective, the identified habitat-specific core functional microbes can guide the development of site-specific microbial inoculants, which help improve soil microenvironment suitability, boost plant growth, and stabilize the accumulation of bioactive metabolites in artificially planted *C. spinosa* to meet clinical quality standards. For pharmacological research, the observed associations between rhizosphere microbial taxa and the accumulation of active components suggest a research direction for exploring microbial biosynthetic pathways of bioactive compounds, laying a foundation for improving the yield of effective pharmacological components through microbial metabolic engineering.

## 5 Conclusion

This study found that habitat was associated with the differences in the relative fruit metabolite profile of *C. spinosa* and in the rhizosphere soil metabolite and microbial community composition. Across the three habitats, Urumqi fruits generally showed the highest relative levels of the selected candidate quality-associated metabolites, while Karamay fruits generally showed the lowest, with the soil nutrient-related metabolites following a broadly similar pattern. Correlation analyses identified candidate soil metabolites and microbial taxa associated with these fruit metabolites, pointing to habitat, soil chemistry, and the rhizosphere microbiome as interconnected factors worth further investigation. However, as this is an observational, correlation-based study, these relationships do not establish causal regulation of metabolite accumulation, and Urumqi should not be regarded as a demonstrably optimal cultivation region without a controlled cultivation experiment. These findings are based on relative (semi-quantitative) LC-MS responses and putative metabolite annotations rather than absolute quantification or authentic-standard confirmation. Targeted quantitative validation, standard-based identification of key compounds, and controlled cultivation or manipulation experiments are therefore needed before habitat-based cultivation or microbiome-management recommendations can be made.

## Data Availability

The 16S rDNA Illumina libraries obtained from the sequencingcompany were deposited at the NCBI’s small read archive (SRA) inBioProject ID: PRJNA1259189.

## References

[B1] Abreu D. N. I. Mazzafera P. (2005). Effect of water and temperature stress on the content of active constituents of Hypericum brasiliense Choisy. Plant Physiol. Biochem. 43, 241–248. doi: 10.1016/j.plaphy.2005.01.020 15854832

[B2] Aichi-Yousfi H. Bahri A. B. Medini M. Rouz S. Rejeb N. M. Ghrabi-Gammar Z. (2016). Genetic diversity and population structure of six species of Capparis in Tunisia using AFLP markers. C.R. Biol. 339, 442–453. doi: 10.1016/j.crvi.2016.09.001 27697367

[B3] Amal A. Mohamad K. Giuseppe P. Nadia M. (2019). Quaternary ammonium compounds in roots and leaves of Capparis spinosa L. from Saudi Arabia and Italy: investigation by HPLC-MS and ¹H NMR. Nat. Prod. Res. 33, 1322–1328. doi: 10.1080/14786419.2018.1475376 29781300

[B4] Anna K. Agnieszka K. Jacek P. Andrzej S. Jarosław G. Anna M. . (2023). Bacteroidota structure in the face of varying agricultural practices as an important indicator of soil quality – a culture independent approach. Agric. Ecosyst. Environ. 342. doi: 10.1016/j.agee.2022.10825242574925

[B5] Asaf S. Numan M. Khan A. L. Al-Harrasi A. (2020). Sphingomonas : from diversity and genomics to functional role in environmental remediation and plant growth. Crit. Rev. Biotechnol. 40, 138–152. doi: 10.1080/07388551.2019.1709793 31906737

[B6] Asghari B. Khademian R. Sedaghati B. (2020). Plant growth promoting rhizobacteria (PGPR) confer drought resistance and stimulate biosynthesis of active components in pennyroyal (Mentha pulegium L.) under water shortage condition. Sci. Hortic. 263, 109132. doi: 10.1016/j.scienta.2019.10913242574925

[B501] Ayu M. Reka S. M. Ayun A. D. Budiman Y. Nur R. R. Talha E. B. . (2022). Terpenes and terpenoids as main bioactive compounds of essential oils, their roles in human health and potential application as natural food preservatives. Food Chem. X 13, 100217. doi: 10.1016/j.fochx.2022.100217 PMC9039924 35498985

[B7] Azzurra S. Gokhan Z. Marcello L. Giorgia M. Kun C. W. Ettore N. . (2018). Impact of different geographical locations on varying profile of bioactives and associated functionalities of caper (Capparis spinosa L.). Food Chem. Toxicol. 118, 181–189. doi: 10.1016/j.fct.2018.05.003 29751072

[B8] Badri D. V. Vivanco J. M. (2009). Regulation and function of root exudates. Plant Cell Environ. 32, 666–681. doi: 10.1111/j.1365-3040.2008.01926.x 19143988

[B9] Basile A. Riggio P. F. Tescari M. Chebbi A. Sodo A. Bartoli F. . (2025). Metagenome-resolved functional traits of Rubrobacter species implicated in rosy discoloration of ancient frescoes in two Georgian Cathedrals. Sci. Total Environ. 958, 178135. doi: 10.1016/j.scitotenv.2024.178135 39705954

[B10] Bovio W. P. Guerrero L. D. Erijman L. Oyarzúa P. SuárezOjeda M. E. Cabezas A. . (2023). Genome-centric metagenomic insights into the role of Chloroflexi in anammox, activated sludge and methanogenic reactors. BMC Microbiol. 23, 45–45. doi: 10.1186/S12866-023-02765-5 36809975 PMC9942424

[B11] Che J. L. Wu Y. Q. Chang Y. Wu W. L. Lyu L. F. Cao F. L. . (2026). Significant benefits of the rhizosphere microbiome for sustainable agriculture: a review on blueberry rhizosphere microbiome. Agronomy 16, 718–718. doi: 10.3390/AGRONOMY1607071830654563

[B12] Chen A. Mao X. Sun Q. H. Wei Z. X. Li J. You Y. L. . (2021). Alternaria mycotoxins: An overview of toxicity, metabolism, and analysis in food. J. Agric. Food. Chem. 69. doi: 10.1021/ACS.JAFC.1C03007 34250809

[B13] Chen D. Feng Q. Y. Liang H. Q. (2021). Effects of long-term discharge of acid mine drainage from abandoned coal mines on soil microorganisms: microbial community structure, interaction patterns, and metabolic functions. Environ. Sci. pollut. Res. Int. 28, 53936–53952. doi: 10.1007/S11356-021-14566-2 34046832

[B14] Cheng N. Peng Y. J. Kong Y. L. Li J. J. Sun C. X. (2018). Combined effects of biochar addition and nitrogen fertilizer reduction on the rhizosphere metabolomics of maize (Zea mays L.) seedlings. Plant Soil 433, 19–35. doi: 10.1007/s11104-018-3811-630311153

[B16] Devin C. Damaris D. Citlali F. Stephen G. Scott C. Tanja W. . (2016). Plant compartment and biogeography affect microbiome composition in cultivated and native Agave species. New Phytol. 209, 798–811. doi: 10.1111/nph.13697 26467257 PMC5057366

[B17] Ding J. Sun H. W. Liang A. P. Liu J. Song L. H. Lv M. . (2020). Testosterone amendment alters metabolite profiles of the soil microbial community. Environ. pollut. 272, 115928. doi: 10.1016/j.envpol.2020.115928 33139098

[B18] Dong C. B. Shao Q. Y. Ran Q. S. Li X. Han Y. F. (2024). Interactions of rhizosphere microbiota-environmental factors-pharmacological active ingredients of Eucommia ulmoides. Planta 259, 59–59. doi: 10.1007/S00425-024-04338-W 38311641

[B19] Duan D. D. Jiang F. F. Lin W. H. Tian Z. Wu N. N. Feng X. X. . (2022). Effects of drought on the growth of Lespedeza davurica through the alteration of soil microbial communities and nutrient availability. J. Fungi 8, 384. doi: 10.3390/JOF8040384 35448615 PMC9025084

[B15] Dunn W. B. Broadhurst D. Begley P. Zelena E. Francis-McIntyre S. Anderson N. . (2011). Procedures for large-scale metabolic profiling of serum and plasma using gas chromatography and liquid chromatography coupled to mass spectrometry. Nat. Protoc. 6, 1060–1083. doi: 10.1038/nprot.2011.335 21720319

[B21] Fotios T. Zoi S. Periklis V. Despina K. C. G. M. Konstantinos P. . (2023). Redox biomarkers assessment after oral administration of wine extract and grape stem extract in rats and mice. Molecules 28, 1574. doi: 10.3390/MOLECULES28041574 36838560 PMC9965357

[B22] Gao F. G. Jiao S. Dong K. Liu X. Xin X. Chen B. B. . (2026). Soil microbial biogeography: from maps to blueprints. Soil Ecol. Lett. 8, 260423–260423. doi: 10.1007/S42832-026-0423-630311153

[B23] Gianguzzi V. Inglese P. Barone E. Sottile F. (2019). *In vitro* regeneration of Capparis spinosa L. by using a temporary immersion system. Plants 8, 177–177. doi: 10.3390/plants8060177 31208122 PMC6630581

[B24] Gu Y. Wang J. H. Cai W. J. Li G. L. Mei Y. Yang S. H. (2021). Different amounts of nitrogen fertilizer applications alter the bacterial diversity and community structure in the rhizosphere soil of sugarcane. Front. Microbiol. 12, 721441. doi: 10.3389/fmicb.2021.721441 34616383 PMC8489880

[B25] Gustavo S. (2022). How plants recruit their microbiome? New insights into beneficial interactions. J. Adv. Res. 40, 45–58. doi: 10.1016/J.JARE.2021.11.020 36100333 PMC9481936

[B27] Hassan A. Yaya S. M. T. G. B. Widad B. B. Badreddine D. Ismail M. . (2022). Caper (Capparis spinosa L.): An updated review on its phytochemistry, nutritional value, traditional uses, and therapeutic potential. Front. Pharmacol. 2022, 13878749–878749. doi: 10.3389/FPHAR.2022.878749 35935860 PMC9353632

[B28] Hassanzadeh A. Hamidpour M. Dahaji A. P. Akhgar A. Khalil K. (2024). Maximizing the efficiency of layered double hydroxides as a slow-release phosphate fertilizer: A study on the impact of plant growth-promoting rhizobacteria. Appl. Clay Sci. 262, 107620. doi: 10.1016/J.CLAY.2024.10762042574925

[B29] He C. Han T. T. Liu C. Sun P. Liao D. Q. Li X. E. (2023). Deciphering the effects of genotype and climatic factors on the performance, active ingredients and rhizosphere soil properties of Salvia miltiorrhiza. Front. Plant Sci. 14, 1110860. doi: 10.3389/FPLS.2023.1110860 37152152 PMC10157250

[B30] He X. L. Li Q. Peng W. H. Zhou J. Cao X. L. Wang D. . (2017). Intra- and inter-isolate variation of ribosomal and protein-coding genes in Pleurotus: implications for molecular identification and phylogeny on fungal groups. BMC Microbiol. 17, 139. doi: 10.1186/s12866-017-1046-y 28651582 PMC5485676

[B31] Hu D. B. Zhang S. Li M. Wu X. X. (2017). Chemical constituents of Capparis spinosa. Chem. Nat. Compd. 53, 557–558. doi: 10.1007/s10600-017-2048-630311153

[B33] Hua D. W. Li J. Xu Y. (2020). Influence of topographical factors on spatial distribution characteristics of soil nutrients in Qinba Mountain Area. IOP Conf. Series: Earth Environ. Sci. 558, 32025. doi: 10.1088/1755-1315/558/3/032025

[B32] Hua C. Yulu J. Shenchong L. Lin L. Min T. Wei Y. . (2019). Extensive metabolic profiles of leaves and stems from the medicinal plant Dendrobiumofficinale Kimura et Migo. Metabolites 9, 215–215. doi: 10.3390/metabo9100215 31590300 PMC6835975

[B34] Huang B. Chen Y. X. Cao Y. Liu D. Y. Fang H. Zhou C. C. . (2024). The structure and function of rhizosphere bacterial communities: impact of chemical vs. bio-organic fertilizers on root disease, quality, and yield of Codonopsis pilosula. Front. Microbiol. 15, 1484727. doi: 10.3389/FMICB.2024.1484727 39498142 PMC11532114

[B35] Huseini F. H. Hasani-Rnjbar S. Nayebi N. Heshmat R. Sigaroodi K. F. Ahvazi A. M. . (2013). Capparis spinosa L. (Caper) fruit extract in treatment of type 2 diabetic patients: A randomized double-blind placebo-controlled clinical trial. Complementary Therapies Med. 21, 447–452. doi: 10.1016/j.ctim.2013.07.003 24050578

[B37] Ji P. Yang X. Q. Zhao X. S. (2024). Application of metabolomics in quality control of traditional Chinese medicines: a review. Front. Plant Sci. 15, 1463666. doi: 10.3389/FPLS.2024.1463666 39619843 PMC11604444

[B39] Jiang W. Jiang L. L. Yin X. L. Zhang S. H. Duan X. J. Chen J. D. . (2025). Untargeted metabolomics reveals the metabolic characteristics and biomarkers of antioxidant properties of Gardeniae Fructus from different geographical origins in China. Metabolites 15, 38. doi: 10.3390/metabo15010038 39852381 PMC11767249

[B38] Jiang S. Lu H. L. Zhang Q. Liu J. C. Yan C. L. (2016). Effect of enhanced reactive nitrogen availability on plant-sediment mediated degradation of polycyclic aromatic hydrocarbons in contaminated mangrove sediment. Mar. pollut. Bull. 103, 151–158. doi: 10.1016/j.marpolbul.2015.12.027 26749225

[B40] Juan'e D. Liang Z. S. Wei Q. Chen S. L. (2006). Effective constitutents contents in indigowood roots and leaves from different regions. Ying yong sheng tai xue bao = J. Appl. Ecol. 17, 1613–1618. 17147167

[B41] Kabir S. M. Wazed A. M. Rahman M. M. Akter S. M. Ahmed M. (2024). Metabolomics of parboiled and non-parboiled rice on metabolites, antioxidant capacity, and minerals content in raw rice, cooked rice, and rice gruel. J. Food Meas. Charact. 18, 6551–6560. doi: 10.1007/S11694-024-02670-Z30311153

[B45] Li Y. Q. Kong D. X. Fu Y. Suaaman R. M. Wu H. (2020). The effect of developmental and environmental factors on secondary metabolites in medicinal plants. Plant Physiol. Biochem. 148, 80–89. doi: 10.1016/j.plaphy.2020.01.006 31951944

[B42] Li L. L. Li Q. F. (2026). Analysis of rhizosphere soil bacterial community structure and diversity in Acanthopanax giraldiifrom different regions of Qinghai. Forum Res. Innovation Manage. 4. doi: 10.70711/FRIM.V4I3.8751

[B44] Li Y. K. Zou N. Liang X. J. Zhou X. Guo S. H. Wang Y. J. . (2023). Effects of nitrogen input on soil bacterial community structure and soil nitrogen cycling in the rhizosphere soil of Lycium barbarum L. Front. Microbiol. 13, 1070817. doi: 10.3389/fmicb.2022.1070817 36704567 PMC9871820

[B49] Liu X. J. Kong F. D. Xiao N. Li X. Y. Zhang M. Y. Lv F. J. . (2023). Prenylated indole-terpenoids with antidiabetic activities from Penicillium sp.HFF16 from the rhizosphere soil of Cynanchum bungei Decne. Front. Microbiol. 14, 1099103. doi: 10.3389/fmicb.2023.1099103 36937284 PMC10018213

[B48] Liu S. J. Min X. Y. Li J. Y. Song W. Liu Z. X. Li X. J. . (2026). Long-term succession and driving mechanisms of soil microbial communities in reclaimed farmland. Catena 266, 109952. doi: 10.1016/j.catena.2026.10995242574925

[B47] Liu J. Ye L. Jing C. Y. (2022). Active microbial arsenic methylation in saline-alkaline paddy soil. Sci. Total Environ. 865, 161077. doi: 10.1016/j.scitotenv.2022.161077 36572312

[B50] Ma H. C. Chu Z. J. Shi F. X. Liu Q. C. Yao Q. X. (2016). Effects of enhanced UV-B radiation on the nutritional and active ingredient contents during the floral development of medicinal chrysanthemum. J. Photochem. Photobiology B. Biol. 158, 228–234. doi: 10.1016/j.jphotobiol.2016.02.019 26985737

[B51] Manici M. L. Caputo F. Sabata D. D. Fornasier F. (2024). The enzyme patterns of Ascomycota and Basidiomycota fungi reveal their different functions in soil. Appl. Soil Ecol. 196, 105323. doi: 10.1016/j.apsoil.2024.10532342574925

[B52] Marchev A. S. Vasileva L. V. Amirova K. M. Savova M. S. BalchevaSivenova Z. P. Georgiev M. I. (2021). Metabolomics and health: from nutritional crops and plant-based pharmaceuticals to profiling of human biofluids. Cell. Mol. Life. Sci. CMLS 78, 1–17. doi: 10.1007/s00018-021-03918-3 34410445 PMC8558153

[B53] Mauceri A. Puccio G. Faddetta T. Abbate L. Polito G. Caldiero C. . (2024). Integrated omics approach reveals the molecular pathways activated in tomato by Kocuria rhizophila, a soil plant growth-promoting bacterium. Plant Physiol. Biochem. PPB 210, 108609. doi: 10.1016/j.plaphy.2024.108609 38615442

[B54] Meng T. T. Zhao T. T. Leng F. F. Chen J. X. Wang Y. G. (2023). Changes of key soil factors, biochemistry and bacterial species composition during seasons in the rhizosphere and roots of Codonopsis pilosula (tangshen). Agronomy 13. doi: 10.3390/agronomy1306154530654563

[B55] Miao S. J. Qiao Y. F. Jin J. Wang Y. H. Tang C. X. (2020). Greater variation of bacterial community structure in soybean- than maize-grown Mollisol soils in responses to seven-year elevated CO_2_ and temperature. Sci. Total Environ. 764, 142836. doi: 10.1016/j.scitotenv.2020.142836 33160664. p.

[B56] Ming Q. L. Su C. Y. Zheng C. Jia M. Zhang Q. Y. Zhang H. . (2013). Elicitors from the endophytic fungus Trichoderma atroviride promote Salvia miltiorrhiza hairy root growth and tanshinone biosynthesis. J. Exp. Bot. 64, 5687–5694. doi: 10.1093/jxb/ert342 24127517

[B57] Moreira A. Moraes L. A. C. (2017). Yield, nutritional status and soil fertility cultivated with common bean in response to amino-acids foliar application. J. Plant Nutr. 40, 344–351. doi: 10.1080/01904167.2016.124019437339054

[B58] Mu M. J. Zhang D. G. Zhang H. Yang M. Guo D. Q. Zhou N. (2019). Correlation between rhizospheric microorganisms distribution and alkaloid content of Fritillaria taipaiensis. Zhongguo Zhong yao za zhi = Zhongguo zhongyao zazhi = China J. Chin. materia Med. 44, 2231–2235. doi: 10.19540/j.cnki.cjcmm.20190301.013 31359647

[B59] Muhammad K. Rahman U. S. Muhammad B. Feng D. H. (2018). Role of flavonoids in plant interactions with the environment and against human pathogens — A review. J. Integr. Agric. 18, 211–230. doi: 10.1016/S2095-3119(19)62555-4

[B60] Naghiloo S. Movafeghi A. Delazar A. Nazemiyeh H. Asnaashari S. Dadpour R. M. (2012). Ontogenetic variation of total phenolics and antioxidant activity in roots, leaves and flowers of Astragalus compactus Lam. (Fabaceae). BioImpacts 2, 105–109. doi: 10.5681/BI.2012.015 23678448 PMC3648924

[B61] Norese A. C. Maldonado E. J. Hodar C. Galvez G. Palma E. D. Cambiazo V. . (2025). Ironing out the conflicts: iron supplementation reduces negatives bacterial interactions in the rhizosphere of an Atacama-endemic perennial grass. Environ. Microbiome 20, 29. doi: 10.1186/S40793-024-00661-7 40069904 PMC11899425

[B62] Nowruzian A. Aalami A. (2025). Physiological, biochemical, and transcriptional responses under drought stress in Capparis spinosa L. Russ. J. Plant Physiol. 72, 233–233. doi: 10.1134/s1021443725604355

[B63] Osei K. A. Thevathasan V. N. Oelbermann M. (2025). Soil organic carbon storage of different soil‐sized fractions in perennial bioenergy crops on marginally productive cropland in Southern Canada. GCB Bioenergy 17, e70025–e70025. doi: 10.1111/GCBB.7002540046247

[B64] Özcan M. M. Efe S. N. (2024). Effect of fermentation on chemical and bioactive properties and phenolic profiles of caper (Capparis ovata Desf. var. ovata) flower buds in three different sizes. J. Saudi Soc Agric. Sci. 23, 74–87. doi: 10.1016/j.jssas.2023.09.00442574925

[B65] Pawlik M. Płociniczak T. Thijs S. Pintelon I. Vangronsveld J. Piotrowska-Segetet Z. (2020). Comparison of two inoculation methods of endophytic bacteria to enhance phytodegradation efficacy of an aged petroleum hydrocarbons polluted soil. Agronomy 10, 1196. doi: 10.3390/agronomy1008119630654563

[B66] Peng Z. Guo X. Z. Xu Y. Liu D. H. Wang H. Y. Guo L. P. . (2020). Advances in interaction between medicinal plants and rhizosphere microorganisms. Zhongguo Zhong yao za zhi = Zhongguo zhongyao zazhi = China J. Chin. materia Med. 45, 2023–2030. doi: 10.19540/j.cnki.cjcmm.20200302.116 32495548

[B67] Pérez S. A. C. Jiménez M. M. S. Jiménez M. M. H. Morales R. Y. Batista A. R. Marrero R. A. . (2026). Revealing and characterizing bacterial communities of *in vitro* Musa species through 16S rDNA metabarcoding and culture dependent approaches. Sci. Rep. 16, 5214–5214. doi: 10.1038/S41598-026-35510-9 41535415 PMC12881421

[B69] Sadhana S. Pushplata S. Shruti S. Sajad A. Nidhi D. (2023). Scope of onsite, portable prevention diagnostic strategies for Alternaria infections in medicinal plants. Biosensors 13. doi: 10.3390/BIOS13070701 37504100 PMC10377195

[B70] Sidhu D. Vasundhara M. Dey P. (2024). Chemical characterization, pathway enrichments and bioactive potentials of catechin-producing endophytic fungi isolated from tea leaves. RSC Adv. 14, 33034–33047. doi: 10.1039/D4RA05758A 39434990 PMC11492194

[B71] Song S. S. Yang X. Tang R. Zhang Y. Q. Tang Z. Y. (2025). Soil properties and plant functional traits have different importance in shaping rhizosphere soil bacterial and fungal communities in a meadow steppe. mSystems 2025, e0057025. doi: 10.1128/MSYSTEMS.00570-25 40626779 PMC12363235

[B73] Sun X. Dong Y. H. Wang N. Cui W. H. Liao X. Y. Liu L. (2020). Screening and evaluation of saline-alkali-tolerant and growth-promoting bacteria. Sheng wu gong cheng xue bao = Chin. J. Biotechnol. 36, 1356–1364. doi: 10.13345/j.cjb.190519 32748593

[B72] Sun R. Guo F. R. Zhang Y. N. Shao H. Yang X. W. Wang C. P. . (2025). Correction: Biological activity of secondary metabolites of actinomycetes and their potential sources as antineoplastic drugs: a review. Front. Microbiol. 16, 1672871. doi: 10.3389/fmicb.2025.1672871 40862139 PMC12372619

[B75] Tripathi A. Pandey P. Tripathi N. S. Kalra A. (2025). Plant growth-promoting endophytic consortium improved artemisinin biosynthesis via modulating antioxidants, gene expression, and transcriptional profile in Artemisia annua (L.) under stressed environments. Plant Stress 15, 100757. doi: 10.1016/j.stress.2025.10075742574925

[B76] Valls J. Richard T. Trotin F. Monti P. J. Mérillon M. J. Vitrac X. (2006). Carbon-14 biolabeling of flavanols and chlorogenic acids in Crataegus monogyna cell suspension cultures. Food Chem. 105, 879–882. doi: 10.1016/j.foodchem.2006.11.06542574925

[B77] Venkatasai N. N. Shetty N. D. Vinay M. C. Sekar M. Muthesamy A. Rai S. P. (2025). A comprehensive review of factors affecting growth and secondary metabolites in hydroponically grown medicinal plants. Planta 261, 48–48. doi: 10.1007/S00425-025-04619-Y 39883244 PMC11782463

[B78] Wang F. Jia M. Y. Li K. Cui Y. F. An L. Z. Sheng H. M. (2024). Sphingomonas sp. Hbc-6 alters Arabidopsis metabolites to improve plant growth and drought resistance by manipulating the microbiome. Microbiol. Res. 287, 127852. doi: 10.1016/J.MICRES.2024.127852 39084119

[B79] Wen B. Mei Z. L. Zeng C. W. Liu S. Q. (2017). metaX: a flexible and comprehensive software for processing metabolomics data. BMC Bioinf. 18, 183. doi: 10.1186/s12859-017-1579-y 28327092 PMC5361702

[B80] Weston L. A. Mathesius U. (2013). Flavonoids: their structure, biosynthesis and role in the rhizosphere, including allelopathy. J. Chem. Ecol. 39, 283–297. doi: 10.1007/s10886-013-0248-5 23397456

[B81] Xia M. X. McCormack L. M. Suseela V. Kennedy G. P. Tharayil N. (2024). Formations of mycorrhizal symbiosis alter the phenolic heteropolymers in roots and leaves of four temperate woody species. New Phytol. 242, 1476–1485. doi: 10.1111/NPH.19731 38659127

[B82] Xie Y. F. Lu Y. X. Jiang X. M. Hu S. X. Fu J. Y. Pan P. . (2025). Integrated metabolome and transcriptome analysis revealed the mechanism of secondary metabolite biosynthesis in Bletilla striata under UV-C treatment. Plant Physiol. Biochemistry: PPB 230, 110892. doi: 10.1016/j.plaphy.2025.110892 41380622

[B83] Xu N. Tan G. C. Wang H. Y. Gai X. P. (2016). Effect of biochar additions to soil on nitrogen leaching, microbial biomass and bacterial community structure. Eur. J. Soil Biol. 74, 1–8. doi: 10.1016/j.ejsobi.2016.02.00442574925

[B87] Yang X. Lai J. L. Zhang Y. Luo X. G. Han M. W. Zhao S. P. (2021). Microbial community structure and metabolome profiling characteristics of soil contaminated by TNT, RDX, and HMX. Environ. pollut. (Barking Essex: 1987) 285, 117478. doi: 10.1016/j.envpol.2021.117478 34087636

[B88] Yang Y. Li T. Wang Y. Q. Cheng H. Chang S. X. Liang C. . (2021). Negative effects of multiple global change factors on soil microbial diversity. Soil Biol. Biochem. 156. doi: 10.1016/j.soilbio.2021.10822942574925

[B85] Yang L. L. Yang L. Lan Y. M. Zhao Y. Han M. Yang M. (2020). Exogenous abscisic acid reduces saikosaponin accumulation by inhibiting saikosaponin synthesis pathway gene expression under drought stress in Bupleurum chinense DC. Ind. Crops Prod. 154, 112686. doi: 10.1016/j.indcrop.2020.11268642574925

[B500] You H. Yang S. J. Zhang L. Hu X. F. Li Q. (2018). Promotion of phenolic compounds production in Salvia miltiorrhiza hairy roots by six strains of rhizosphere bacteria. Eng. Life Sci. 18 (3), 160–168. doi: 10.1002/elsc.201700077 PMC6999580 32624895

[B90] Yuan C. X. Ma Z. X. Liu S. Y. Nie H. L. Feng G. Z. Wang S. J. . (2025). Effects of strip-tillage on soil microbial community structure and function in black soil. Front. Microbiol. 16, 1730920. doi: 10.3389/FMICB.2025.1730920 41503277 PMC12772444

[B93] Zhang Z. G. Jiang H. W. Zhu H. Y. Sun T. G. Xu H. Zhu Q. M. . (2025). Effects of photosynthetic properties, soil environment and bioactive constituents of Eucommia ulmoides Oliver under two planting models. BMC Plant Biol. 25, 826–826. doi: 10.1186/s12870-025-06779-1 40604421 PMC12220568

[B92] Zhang Y. H. Zheng L. L. Zheng Y. Xue S. Zhang J. X. Huang P. . (2020). Insight into the assembly of root-associated microbiome in the medicinal plant Polygonum cuspidatum. Ind. Crops Prod. 145, 112163. doi: 10.1016/j.indcrop.2020.11216342574925

[B94] Zhao C. He X. X. Dan X. Q. Zhao J. Huang X. Q. Cai Z. C. . (2021). Specific dissolved organic matter components drive the assembly of a core microbial community in acidic soil of ammonium-preferring plants. Catena 207. doi: 10.1016/j.catena.2021.10558442574925

[B95] Zhao J. L. Zhou L. G. Wu J. Y. (2010). Promotion of Salvia miltiorrhiza hairy root growth and tanshinone production by polysaccharide–protein fractions of plant growth-promoting rhizobacterium Bacillus cereus. Process Biochem. 45, 1517–1522. doi: 10.1016/j.procbio.2010.05.03442574925

[B96] Zhu Z. P. Chen R. B. Zhang L. (2023). Simple phenylpropanoids: Recent advances in biological activities, biosynthetic pathways, and microbial production. Nat. Prod. Rep 41 (1), 6–24. doi: 10.1039/D3NP00012E 37807808

[B97] Zong T. Q. Li M. X. Hu Z. Y. Jin L. Liu Y. N. Duan Y. Q. . (2025). Traditional uses, phytochemistry, pharmacology, and toxicology of Belamcanda chinensis: a review. Plants 14, 3688. doi: 10.3390/PLANTS14233688 41375398 PMC12693795

[B98] Zou Q. J. Guo Q. S. Wang T. Chen J. M. Yang F. Yang C. L. (2022). Comparison of metabolome characteristics and screening of chemical markers in Chrysanthemum indicum from different habitats. Physiol. Mol. Biol. Plants 28, 65–76. doi: 10.1007/s12298-022-01137-z 35221572 PMC8847665

