# Supplementary material for: Metabolomics-based comparison of metabolite profiles in Capparis spinosa L. from different habitats and associated soil factors
Source: Front Plant Sci. 2026 Oct 14;17:1914134. doi: 10.3389/fpls.2026.1914134 (PMC13715592; doi:10.3389/fpls.2026.1914134)
Supplement: Supplementary file 1 [file DataSheet1.docx]

Fig S1A. Images of *Capparis spinosa* L. fruits from Urumqi, along with field sampling photographs. B. Images of *Capparis spinosa* L. fruits from Karamay, along with field sampling photographs. C. Images of *Capparis spinosa* L. fruits from Ili, along with field sampling photographs.

Fig S2A. For six biological replicate samples of Prickly Ash soil from different habitats, base peak ion current diagrams of the six major peaks were plotted in cation mode. B. For six biological replicates of soil samples from different habitats of Prickly Ash, base peak ion current diagrams of the six major peaks were plotted in negative ion mode.

Fig. S3 The analysis of differential metabolites in fruits of *C. spinosa* from different habitats (negative ion mode). UF, KF, and IF represent Urumqi fruit, Karamay fruit, and Ili fruit, respectively. *p* < 0.05; *p < 0.01;* **p* < 0.001. A. PCA score plots showing the separation among UF, KF, and IF groups. B. OPLS-DA volcano plots displaying differential metabolites (red: upregulated; blue: downregulated; gray: non-significant).

Fig S4A.The PLS-DA ranking validation plot of *C. spinosa* from different habitats (Positive ion mode). B.The PLS-DA ranking validation plot of *C. spinosa* from different habitats (Negative ion mode).“UF”,“KF” and “IF” represent Urumqi fruit, Karamay fruit, Ili fruit respectively. UF (Urumqi fruits), KF (Karamay fruits) an d IF (Ili fruits).

Fig S5A. Base peak ion current plots of the six major peaks were plotted in positive mode for six biological replicates of Zanthoxylum fruits from different habitats. B. Base peak ion current plots of the six major peaks were plotted in negative mode for six biological replicates of Zanthoxylum fruits from different habitats.

Fig. S6 The analysis of differential metabolites in soil of *C. spinosa* from different habitats (negative ion mode). US, KS, and IS represent Urumqi soil, Karamay soil, and Ili soil, respectively. *p* < 0.05; *p < 0.01;* **p* < 0.001. A. PCA score plots showing the separation among US, KS, and IS groups. B. OPLS-DA volcano plots displaying differential metabolites (red: upregulated; blue: downregulated; gray: non-significant).

Fig S7A.The PLS-DA ranking validation plot of *C. spinosa* in Different Habitat (Positive ion mode). B.The PLS-DA ranking validation plot of *C. spinosa* in Different Habitat (Negative ion mode). “US”, “KS” and “IS” represent Urumqi soil, Karamay soil, Ili soil respectively. US (Urumqi soil), KS (Karamay soil) and IS (Ili soil)

Fig S8A. composition of bacterial communities in different habitats at the phylum B. Species composition of fungal communities in different habitats at the phylum.
